# From guest to host: parasite *Cistanche deserticola* shapes and dominates bacterial and fungal community structure and network complexity

**DOI:** 10.1186/s40793-023-00471-3

**Published:** 2023-02-22

**Authors:** Yujing Miao, Xinke Zhang, Guoshuai Zhang, Zhan Feng, Jin Pei, Chang Liu, Linfang Huang

**Affiliations:** 1 Key Laboratory of Chinese Medicine Resources Conservation, State Administration of Traditional Chinese Medicine of the People’s Republic of China, Institute of Medicinal Plant Development, Chinese Academy of Medical Sciences & Peking Union Medical College, Beijing, 100193 China; 2 State Key Laboratory of Southwestern Chinese Medicine Resources, Chengdu University of Traditional Chinese Medicine, Chengdu, 611137 Sichuan China; 3 Jiangxi University of Traditional Chinese Medicine, Nanchang, 330000 Jiangxi China

**Keywords:** *Cistanche deserticola*, *Haloxylon ammodendron*, Bacteria, Fungi, Rhizosphere, Endosphere

## Abstract

**Background:**

Rhizosphere and plant microbiota are assumed to play an essential role in deciding the well-being of hosts, but effects of parasites on their host microbiota have been rarely studied. Also, the characteristics of the rhizosphere and root microbiota of parasites and hosts under parasitism is relatively unknown. In this study, we used *Cistanche deserticola* and *Haloxylon ammodendron* from cultivated populations as our model parasites and host plants, respectively. We collected samples from BULK soil (BULK), rhizosphere soil of *H. ammodendron* not parasitized (NCD) and parasitized (RHA) to study how the parasite influenced the rhizosphere microbiota of the host. We also collected samples from the rhizosphere soil and roots of *C. deserticola* (RCD and ECD) and *Haloxylon ammodendron* (RHA and EHA) to explore the difference between the microbiota of the parasite and its host under parasitism.

**Results:**

The parasite reduced the compositional and co-occurrence network complexities of bacterial and fungal microbiota of RHA. Additionally, the parasite increased the proportion of stochastic processes mainly belonging to dispersal limitation in the bacterial microbiota of RHA. Based on the PCoA ordinations and permutational multivariate analysis of variance, the dissimilarity between microbiota of *C. deserticola* and *H. ammodendron* were rarely evident (bacteria, *R*^*2*^ = 0.29971; fungi, *R*^*2*^ = 0.15631). Interestingly, four hub nodes of *H. ammodendron* in endosphere fungal microbiota were identified, while one hub node of *C. deserticola* in endosphere fungal microbiota was identified. It indicated that *H. ammodendron* played a predominant role in the co-occurrence network of endosphere fungal microbiota. Source model of plant microbiome suggested the potential source percentage from the parasite to the host (bacteria: 52.1%; fungi: 16.7%) was lower than host-to-parasite (bacteria: 76.5%; fungi: 34.3%), illustrating that microbial communication was bidirectional, mainly from the host to the parasite.

**Conclusions:**

Collectively, our results suggested that the parasite *C. deserticola* shaped the diversity, composition, co-occurrence network, and community assembly mechanisms of the rhizosphere microbiota of *H. ammodendron*. Additionally, the microbiota of *C. deserticola* and *H. ammodendron* were highly similar and shared. Our findings on parasite and host microbiota provided a novel line of evidence supporting the influence of parasites on the microbiota of their hosts.

**Supplementary Information:**

The online version contains supplementary material available at 10.1186/s40793-023-00471-3.

## Background

Prokaryotic and eukaryotic microbes dominate the planet, and plants have evolved in an environment that is rich in microbial taxa [1]. During the evolutionary time, plants have established close contact with various microbes that belong to the rhizosphere microbiota [2]. Rhizosphere microbiota is the microbiota in the soil surrounding growing plant roots [3]. The release of specific chemical cues via root exudates is an important way that plants impact the kinds of microbes that become part of their root microbiota [3, 4]. Microorganisms use these root exudates as substrates, leading to increased microbial biomass and activity around the roots, which has become known as the ‘rhizosphere effect’ [5]. The ‘rhizosphere effect’ results in changes in the abundance, diversity and composition of bacterial microbiota. Compared to bulk soil, rhizosphere microbiota displayed lower diversity and more complex co-occurrence networks [6–8]. In addition, the rhizosphere effect has large impacts on plant health, since individuals from the rhizosphere microbiota play an important part in key development processes of plants such as nitrogen fixation, phosphorous solubilization, production of plant growth regulators, and disease protection [9–12].

Bacteria and fungi are the most commonly studied groups in rhizosphere microbiota. Bacteria and fungi often interact in rhizosphere microbiota, which can adjust and control the stability of microbiota [13]. For example, bacteria and fungi formed positive and negative connections in the microbiota of maize at different developmental stages [14]. The diversity and function of rhizosphere fungi are often closely related to root exudates such as proteins and sugars. In addition, many root exudates provide energy for rhizosphere fungi and induce greater densities of rhizosphere fungi [15]. Meanwhile, rhizosphere fungi also affect the chemical composition of medicinal plants [16]. For example, rhizosphere fungal microbiota promoted the accumulation of beneficial substances in medicinal parts. Additionally, some fungi secrete plant growth hormones or other important metabolites directly [17, 18].

Apart from host factors, the assembly and stability of the rhizosphere and plant microbiota are strongly affected by microbe-microbe interactions [19–21]. Co-occurrence network analysis is often used to predict the potential microbial interactions across different habitats [21–23]. In co-occurrence networks, assumed hubs (center taxa) interact with other taxa as often as possible, which are considered mediators and guardians of microbiota. Thus, microbe-microbe interactions are assumed to be an important part of plant microbiome development, host nutrient acquisition and environmental fitness [23, 24]. For example, the maize microbiome research reported that bacterial taxa played a more important role in the microbiota network at the early stage, while fungal taxa did so at the late stage. Xiong et al. identified 39 hub nodes in the rhizosphere microbiota of crops [25]. However, knowledge of bacterial-bacterial or fungal-fungal interactions along the parasite-host system is lacking. How these interactions respond to changes across plant richness (hosts and parasites) and the extent to which these complex microbiota interactions affect microbiota dynamics and host performance have not been systematically studied.

*Cistanche deserticola* and *Haloxylon ammodendron* are common desert plants in northwest China, with the ecological effects of wind protection and sand fixation [26]. *C. deserticola* is parasitic upon *H. ammodendron* [27], with economic benefits as both medicine and food. Several studies reported that the pharmacological effects of *Cistanche* included neuroprotective, immunomodulatory, anti-senescence, anti-inflammatory, anti-osteoporosis, hepatoprotective, anti-oxidative, anti-bacterial, anti-tumor and glucose tolerance improving effects [28, 29]. Our previous research showed that the bacterial microbiota was significantly different among the three ecotypes, and the alpha diversity of the grassland soil microbial community was the highest [30]. And the bacterial and fungal microbiota driving the dynamics in geographic patterns of desert crops were important for large-scale standardization of crops to control desertification [31]. However, few studies were focused on the influence of parasites on the microbiota of the hosts or microbe communication between parasites and hosts. *Cistanche deserticola* and *H. ammodendron* were excellent model systems for studying the dynamic change of microbiota and the complexity of microbe communication.

In this study, we have three goals. Firstly, we intended to illustrate the change in the diversity, composition, co-occurrence network structure and community assembly mechanisms among the rhizosphere microbiota of BULK, NCD and RHA. Secondly, we aimed to compare the diversity, composition, co-occurrence network structure and community assembly mechanisms between the rhizosphere and root microbiota of *C. derseticola* and *H. ammodendron* under parasitism. Thirdly, we tried to predict the potential source of bacterial and fungal microbiota of *C. deseticola* and *H. ammodendron*. Our findings on parasite and host microbiota provide a novel line of evidence supporting the hypothesis that the parasites shape and determine bacterial and fungal community assembly and network complexity.

## Materials and methods

### Sample collection

The field experiments were conducted in Alxa, Inner Mongolia Province (38°47′3″ N,105°21′60″ E; northwest China). The ‘Chinese Cistanche planting’ base has a planting history of over 20 years. The soil properties of the base were similar, and unified management of the base was implemented. Cultivated *C. desertcola* and* H. ammodendron* had the same genetic background (Additional file 1: Fig. S1). Bulk soil (BULK) and rhizosphere soil of *H. ammodendron* not parasitized (NCD) and parasitized (RHA) were collected to explore the influence of parasites on the rhizosphere microbiota of *H. ammodendron* microbiomes. And the rhizosphere soil of *H. ammodendron* (RHA) and *C. deserticola* (RCD) was collected to explore the difference between the microbiota of *C. deserticola* and *H. ammodendron* under parasitism. Roots of *H. ammodendron* (EHA) and *C. deserticola* (ECD) were also collected. Each type of sample had six biological replicates. The sampling method of rhizosphere soil was described by *Edwards* [32]. All samples for high-throughput DNA sequencing were immediately placed in liquid nitrogen after sterilization (only for the endosphere samples), transported to the laboratory on dry ice, and stored at − 80 °C until further processing.

### DNA extraction and sequencing

Genomic DNA was extracted from 0.5 g soil, root and stem samples using the HiPure soil DNA kit (Magen, Guangzhou, China) according to the manufacturer's protocols, respectively. The bacterial 16S rRNA gene V5-V7 region was amplified using primers 799F (5′AACMGGATTAGATACCCKG3′) and 1193R (5′ACGTCATCCCCACCTTCC3′) [33]. The PCR conditions were as follows: 95 °C for 2 min, followed by 27 cycles at 98 °C for 10 s, 62 °C for 30 s, and 68 °C for 30 s, and a final extension at 68 °C for 10 min. Each sample had own barcode, which was an eight-base sequence. The ITS2 region of fungal ITS rRNA was amplified with the primers ITS3_KYO2(5′GATGAAGAACGYAGYRAA3′) and ITS4 (5′ TCCTCCGCTTATTGATATGC3′) [34]. The PCR conditions were the same as the 16S. PCR negative controls consisted of ddH_2_O in place of DNA template. Then, amplicons were purified using the AxyPrep DNA gel extraction kit (Axygen Biosciences, Union City, USA) according to the manufacturer's instructions, and quantified using the ABI Step One Plus real-time PCR system (Life Technologies, Foster City, USA). Finally, purified amplicons were pooled in equimolar amounts and paired-end sequenced (2 × 250 cycles) on an Illumina NovaSeq platform (Illumina Inc., San Diego, USA) following standard protocols. A total of 36 libraries were prepared, with 6 from BULK, 6 from NCD, 6 from RHA, 6 from EHA, 6 from RCD, and 6 from ECD. Together, the libraries yielded 2,218,443 16S rRNA sequences and 2,305,280 ITS rRNA sequences used to generate operational taxonomic unit: groups of sequences that are intended to correspond to taxonomic clades or monophyletic groups (20,293 and 3045 OTU, respectively) (Additional file 2: Tables S1, S2). Rarefaction curves showed a flat trend as the number of sequences increased. It indicated that the detected communities were appropriately sampled for all six samples (Additional file 1: Figs. S2–S5).

### Sequence analyses

Adapters and low-quality reads were included in the raw data, which affected the subsequent assembly and analysis. Thus, the raw data were further filtered using FASTP (https://github.com/OpenGene/fastp) by the following rules: (1) reads containing N10% unknown nucleotides were removed; (2) reads containing b80% bases with quality (Q-value) N 20 were removed to obtain high-quality clean reads. Raw tags were merged by FLASH [35] with a minimum overlap of 10 bp and mismatch error rates of 2%. Raw tags containing noisy sequences were filtered using specific filtering methods to obtain high-quality clean tags. Clean tags were searched against the reference database (http://drive5.com/uchime/uchime_download.html) for reference-based chimera checking using the UCHIME algorithm (http://www.drive5.com/usearch/manual/uchime_algo.html). All chimeric tags detected were removed. Only effective tags were retained for further analysis. Operational taxonomic units (OTU) were clustered using USEARCH (v9.2.64) [36] (at ≥ 97% similarity threshold). In each OTU cluster, the representative sequence was the tag sequence with the highest abundance.

### Statistical analyses

We calculated the alpha diversity of the rhizosphere and roots microbiota of the *C. deserticola* and *H. ammodendron* using the R package ‘Phyloseq’ [37], including Observed richness, Chao1, ACE, Shannon, Simpson, InvSimpson and Fisher. The libraries were normalized for alpha diversity analysis and the reads were about 76, 000 for bacteria and 116, 000 for fungi, respectively. Rarefaction analyses were also performed by the R package ‘Phyloseq’ [37]. For beta diversity, weighted Bray–Curtis and UniFrac distance matrices of the OTU dataset were subjected to principal coordinate analysis (PCoA) and Non-metric multidimensional scaling (NMDS) using the R package ‘Phyloseq’ [37]. To test whether the alpha and beta diversity of both bacterial and fungal microbiota varied significantly among samples, we performed the Wilcox test and PERMANOVA analysis [38]. Differential abundance analysis was performed with the R package ‘DESeq2’ [39] and ‘ALDEx2’ [40] to determine whether the abundance of individual phyla, classes, orders, families, genera and OTU changed among different groups.

### Network construction and analysis

To infer the co-association network, we used a method designed to be robust to the compositional nature of microbiota datasets, Spring [41] from R packages ‘NetCoMi’ v.1.1.0 [42]. The taxa were filtered by highestFreq (400) and totalReads (1000). MeasurePar was made in reference to Connor et al. (nlambda = 100, and rep.num = 30) [43]. Networks were visualized using Gephi [44]. Nodes of the microbiota network were assigned roles by degree and closeness centrality (hub nodes: Degree > 15 and 40; closeness centrality > 0.6). The stability of the microbiota network was characterized by robustness based on natural connectivity to understand whether and how the parasite affects the stability of the networks.

### Inferring community assembly mechanisms and source model of plant microbiome (SMPM)

The relative influences of community assembly processes were assessed by a phylogenetic bin-based null model framework, iCAMP, which was recently reported with substantially improved performance [45]. Briefly, iCAMP divided taxa into different phylogenetic groups (bins) to ensure adequate phylogenetic signals to infer selection from phylogenetic diversity; then, the processes (selection, dispersal, drift, or others) dominating each bin were identified, according to the deviation of observed phylogenetic and taxonomic diversity from random patterns simulated by null models; finally, the relative abundance of bins governed by each process was aggregated to evaluate its influence on entire community assembly. The rarefied OTU table was applied to the ecological null model using R packages “iCAMP” [45]. The significance of differences was calculated based on ‘rand.time’ with 1000 replications.

To infer the microbiota communication between the parasite and the host, we used SourceTracker (v.1.0) [46] based on the Bayesian approach to estimate the sources of bacterial and fungal microbiota in each sample.

## Results

### Parasite influences the diversity of the rhizosphere microbiota of the host

**Figure1:**
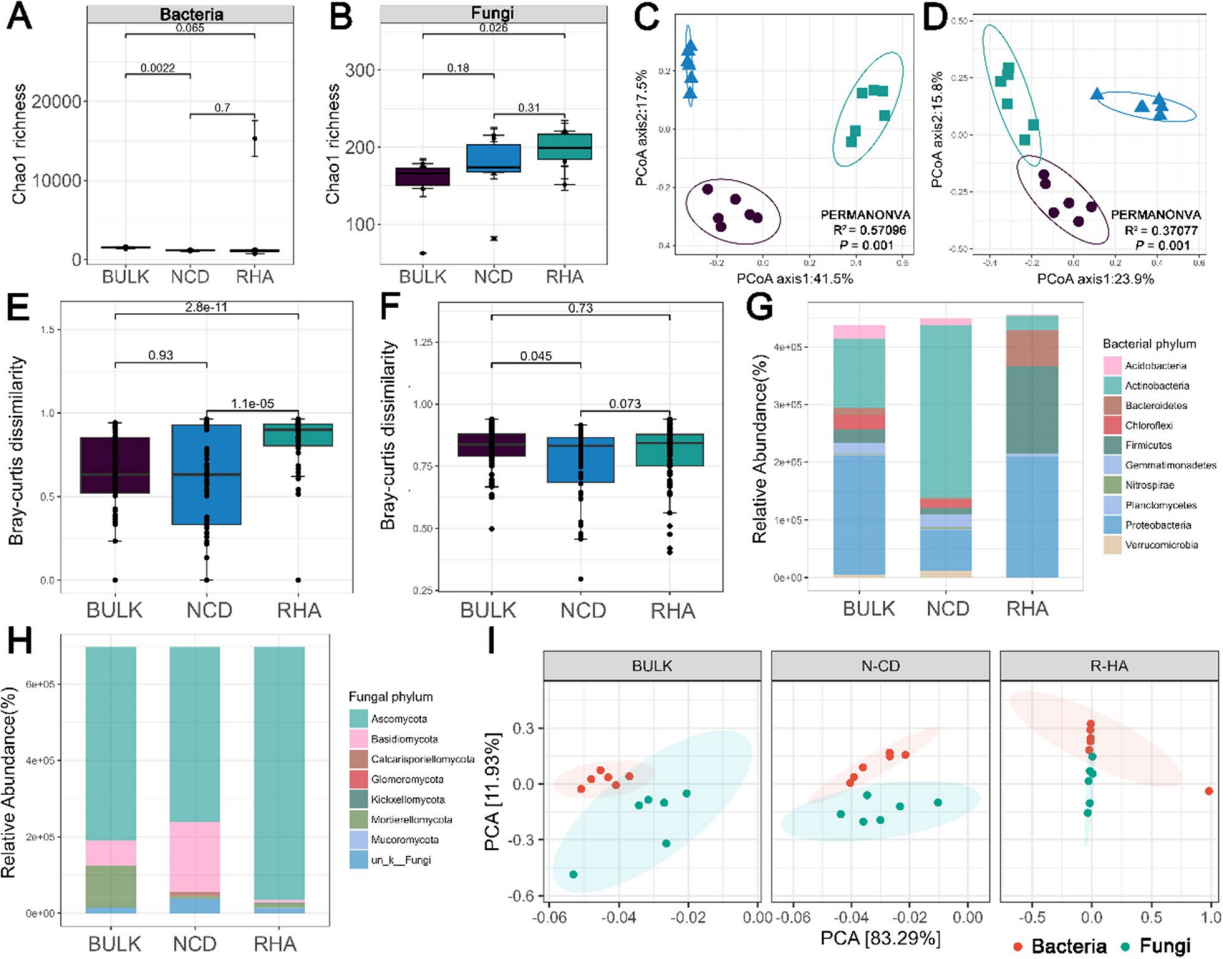
Dynamics of diversity and distribution patterns of bacterial and fungal microbiota across BULK, NCD and RHA. **A** and **B**. Alpha diversity of bacterial (**A**) and fungal (**B**) microbiota. The number on the horizontal line represented *P* value based on Wilcox test. **C** and **D**. Principal-coordinate analysis (PCoA) ordinations based on Bray–Curtis distance matrices describing the distribution patterns of bacterial (**C**) and fungal (**D**) microbiota. The degree of variation explained (R^2^) and the significance (*P* values) provided by permutational multivariate analysis of variance (PERMANOVA) analysis. **E** and **F** Bacterial (**E**) and fungal (**F**) microbiota dissimilarity among three samples (BULK, NCD and RHA). The number on the horizontal line represented *P* value based on Wilcox test. **G** and **H**. The relative abundance of the major microbe phyla in bacterial (**G**) and fungal (**H**) microbiota. **I** Bacterial and fungal microbiota composition dynamics across BULK, NCD and RHA. The percent value for each axis represents the proportion of total variation explained. The ellipses were calculated around barycenters with a confidence level of 0.95 using the stat_conf_ellipse function in ggpubr v.0.2.4

Alpha diversity analyses showed that the rhizosphere bacterial microbiota of *H. ammodendron* parasitized (RHA) was less diverse than that in the rhizosphere bacterial microbiota of *H. ammodendron* not parasitized (NCD) (Fig. 1A; Additional file 1: Fig. S6). And alpha diversity of the rhizosphere bacterial microbiota of NCD was significantly (*P* = 0.0022) less diverse than that in the bacterial microbiota of bulk soil (BULK) (Fig. 1A; Additional file 1: Fig. S6). However, the Chao1 index showed that the richness of the rhizosphere fungal microbiota of RHA was significantly (*P* = 0.026) higher than that in the fungal microbiota of BULK (Fig. 1B). All the alpha diversity indexes declined as the plant richness shifted, indicating that the bacterial microbiota of RHA became simpler in the presence of the parasite (Additional file 1: Fig. S6). On the contrary, the OTU in fungal microbiota became richer in the presence of the parasite (Additional file 1: Fig. S7). The overall pattern of the bulk and rhizosphere soil bacterial and fungal communities of the three samples was delineated on the first two coordinates of principal component analysis (PCoA) and nonmetric multidimensional scaling (NMDS) based on Bray–Curtis and UniFrac distance (Fig. 1C, D; Additional file 1: Figs. S8 and 9). The primary axis of variation (explaining 41.5% of the overall variation) of PCoA separated the microbiota of the BULK, NCD and RHA groups. Permutational multivariate analysis of variance (PERMANOVA) revealed that the presence or absence of host and parasite explained the majority of the variation in bacterial and fungal microbiota (bacteria, *R*^*2*^ = 0.57096, *P* = 0.001; fungi, *R*^*2*^ = 0.37077, *P* = 0.001) (Fig. 1C, D). Bacterial microbiota dissimilarity was significantly higher in the RHA than in the NCD (*P* = 1.1e−05) and BULK (*P* = 2.8e−11) (Fig. 1E). However, the bacterial microbiota dissimilarity between BULK and NCD was not significant (*P* = 0.93). In contrast to bacterial microbiota, the fungal microbiota dissimilarity between RHA and BULK (or NCD) was not significant (Fig. 1F). But the fungal microbiota dissimilarity was significantly lower in the NCD than in the BULK (*P* = 0.045) (Fig. 1F). An ordination plot (Fig. 1I) was used to compare the bacterial and fungal microbiota in the BULK, NCD and RHA samples. Bacterial microbiota overlapped in the BULK and NCD groups, but diverged when parasitized (RHA). In contrast to bacterial microbiota, the fungal microbiota overlapped more in RHA than NCD and BULK (Fig. 1I). Collectively, the dispersion between bacteria and fungi decreased from BULK to NCD to RHA. Relative to the bacterial microbiota of BULK, the bacterial microbiota of NCD exhibited an increased abundance of phyla *Actinobacteria* and a decreased abundance of phyla *Proteobacteria*, while the bacterial microbiota of RHA exhibited an increased abundance of phyla *Firmicutes* and *Bacteroidetes* (Fig. 1G; Additional file 1: Fig. S10). The bacterial microbiota of RHA showed a higher abundance of phyla *Firmicutes*, *Proteobacteria* and *Bacteroidetes* than the bacterial microbiota of NCD (Fig. 1G; Additional file 1: Fig. S10). In fungal microbiota, phylum *Ascomycota* showed high abundance in all the BULK, NCD and RHA groups (Fig. 1H; Additional file 1: Fig. S10). Relative to the fungal microbiota of RHA, the fungal microbiota of NCD exhibited an increased abundance of phylum *Basidiomycota* and the fungal microbiota of BULK exhibited an increased abundance of phyla *Mortierellomycota* and *Basidiomycota* (Fig. 1H; Additional file 1: Fig. S10).

To identify groups that became significantly more prevalent during the dynamic process of the development of parasitism (BULK vs NCD, BULK vs RHA and NCD vs RHA), the differential abundance of the detected bacterial and fungal groups was calculated using Deseq2 and ALDEx2 (Additional file 2: Table S3; Additional file 1: Fig. S11). In the bacterial microbiota, we found that the abundance of 59 genera was significantly higher in NCD than that in BULK, including *Rhodanobacter*, *Adhaeribacter*, *Gemmatimonas*, *Herpetosiphon*, *Massilia*, *Herpetosiphon*, *Belnapia*, *Altererythrobacter*, etc. (*P* < 0.05) (Additional file 2: Table S3; Additional file 1: Fig. S11). The genera *Marinobacter*, *Paenibacillus*, *Gracilimonas*, *Alterococcus*, *Owenweeksai*, etc. became significantly more prevalent in the rhizosphere bacterial microbiota of RHA (*P* < 0.05) (Additional file 1: Fig. S11). In the fungal microbiota, we found seven genera that were significantly higher in NCD than that in BULK, including *Aspergillus*, *Calcarisporiella*, *Coniolariella*, *Darksidea*, *Fusarium*, *Simplicillium* and *Tulostoma* (*P* < 0.05) (Additional file 1: Fig. S12). The genera *Articulospora*, *Cyphellophora*, *Hydropisphaera*, *Iodophanus*, *Lecanicillium*, *Leucoagaricus*, *Monosporascus*, *Neocamarosporium*, *Penicillium*, *Sarocladium* and *Scedosporium* were more prevalent in the fungal microbiota of RHA (*P* < 0.05) (Additional file 1: Fig. S12).

### Parasite influences the co-occurrence network of rhizosphere microbiota of the host

**Fig. 2:**
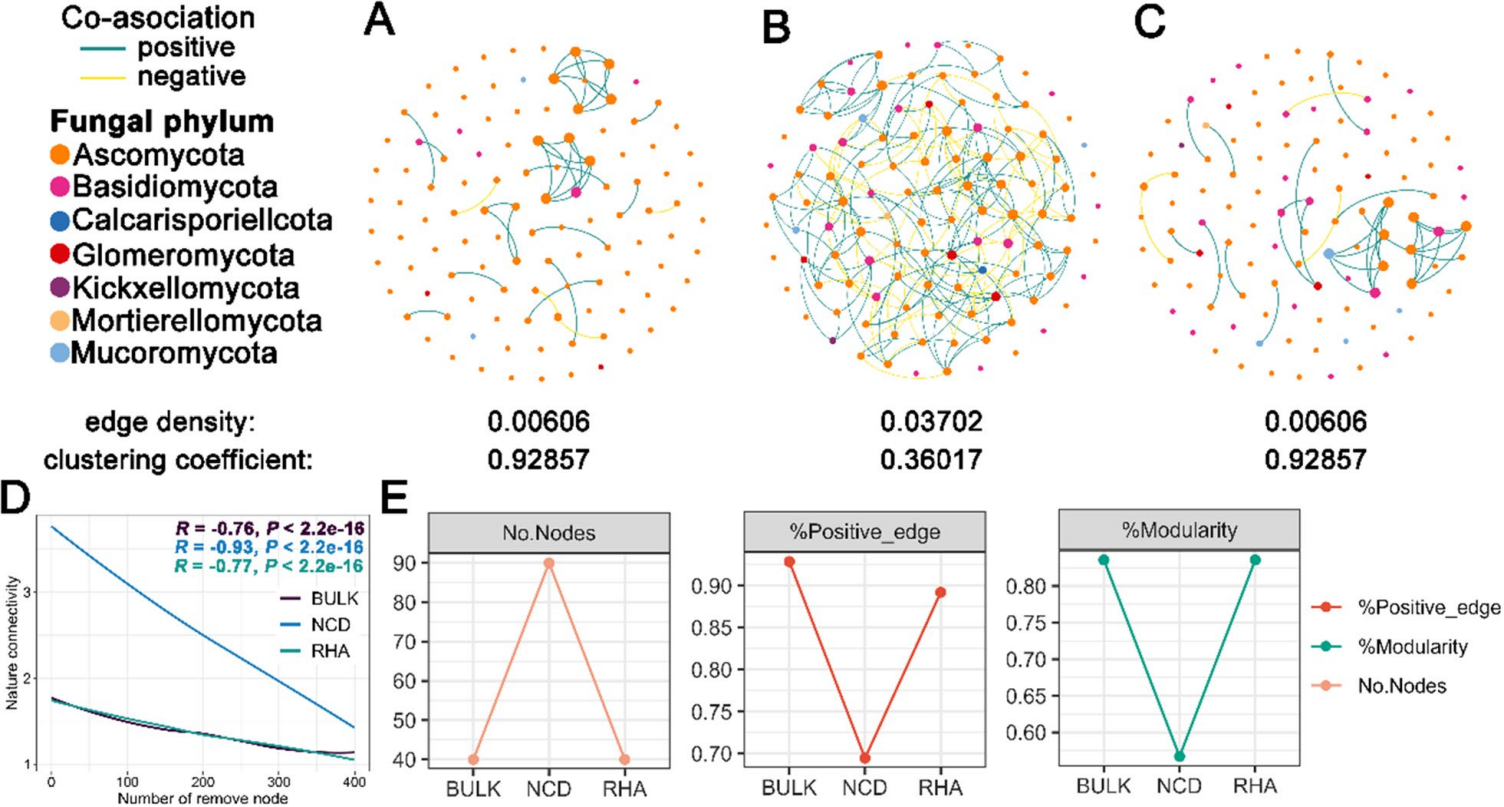
Fungal microbiota networks for BULK, NCD and RHA. **A**, **B** and **C**. Co-occurrence patterns of fungal microbiota networks in BULK (**A**), NCD (**B**) and RHA (**C**). **D** The robustness of microbiota networks was based on natural connectivity. **E** Network topological parameters for fungal microbiota networks

Changes in bacterial and fungal co-occurrence network assembly in the rhizosphere and bulk soil of *H. ammodendron* were characterized by Spring measure-based network analysis. Differences consistently existed in bacterial and fungal microbiota networks during this dynamic change from the absence of plants (BULK) to the presence of the host (NCD) to the presence of the parasite (RHA) (Fig. 2; Additional file 1: Fig. S13). Based on natural connectivity analysis, the highest robustness was observed in the BULK bacterial network (Additional file 1: Fig. S13C). For bacterial microbiota, nodes of co-occurrence networks were gradually increased in RHA (Additional file 1: Fig. S13D). Meanwhile, more connections (larger number of links) and modules were found in the RHA (Additional file 1: Fig. S13D; Additional file 2: Table S4). Conversely, the complexity of the fungal co-occurrence networks was highest in NCD (Fig. 2B). Based on natural connectivity analysis, the highest robustness was observed in the NCD network (Fig. 2C). Moreover, the number of nodes and links showed a consistent trend of change (Fig. 2D; Additional file 2: Table S5). However, the percentage of positive edge and modularity was the lowest in the co-occurrence network of NCD (Additional file 2: Table S5).

### Parasite influences the community assembly mechanisms of the rhizosphere microbiota of the host

**Fig. 3:**
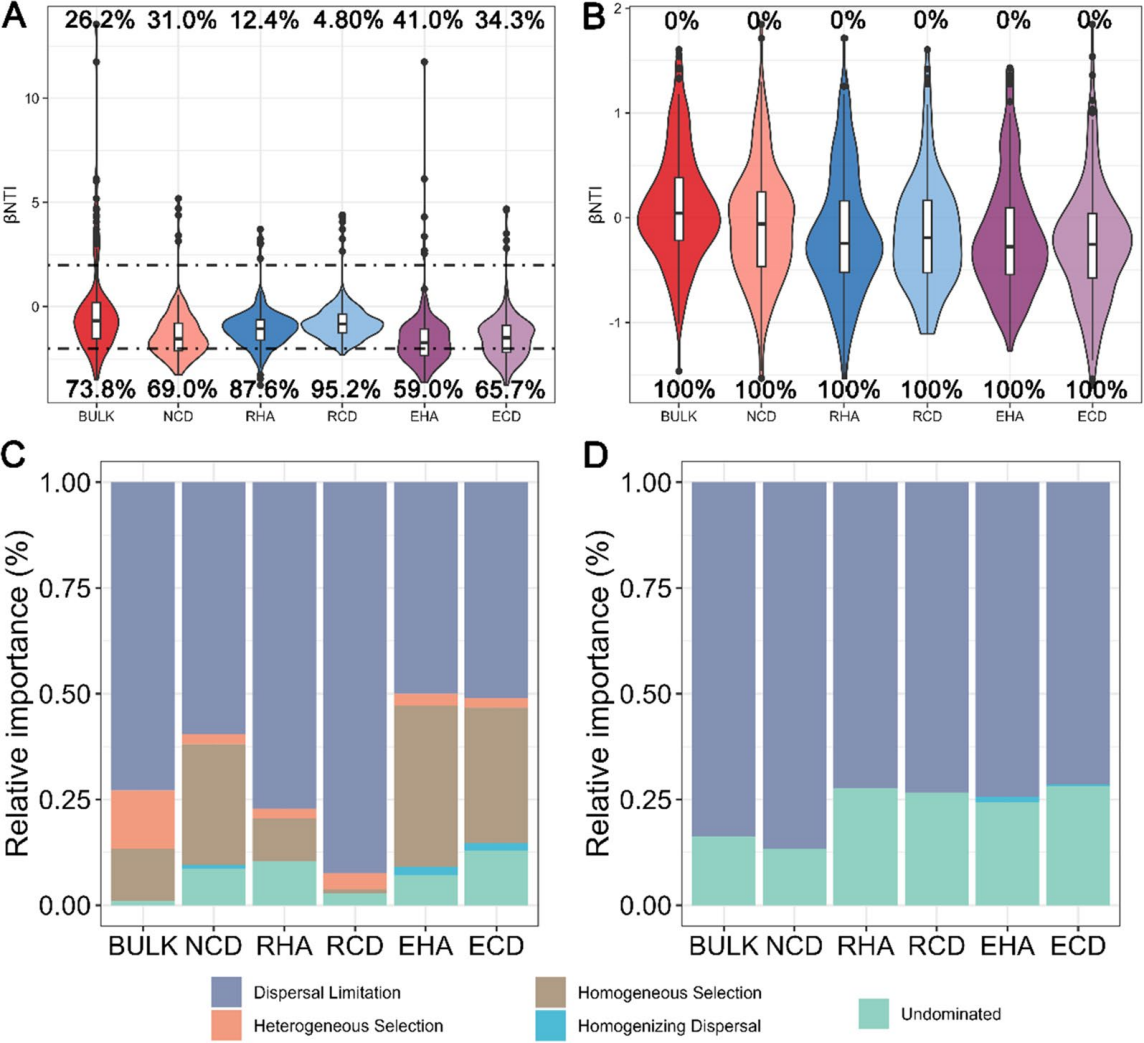
Deterministic and stochastic processes in microbiome assembly. **A** and **B** Relative contribution of determinism and stochasticity on bacterial (**A**) and fungal (**B**) microbiota assembly based on the β-Nearest Taxon Index (βNTI) values. The βNTI values were calculated using null model, and |βNTI|≥ 2 and |βNTI|< 2 represent dominant determinism and stochasticity in driving microbiome assembly, respectively. The percentage above and below the violin plot represent the proportion of the deterministic processes and stochastic processes in microbiome assembly, respectively. **C** and **D**. The relative importance of five ecological processes in bacterial (**C**) and fungal (**D**) microbiota (heterogeneous selection: βNTI <  − 2, homogeneous selection: βNTI >  + 2, dispersal limitation: |βNTI|< 2 and RCBray > 0.95, homogenizing dispersal: |βNTI|< 2 and RCBray < − 0.95, and undominated: |βNTI|< 2 and |RCBray|< 0.95) along the soil–plant continuum based on the β-Nearest Taxon Index (βNTI) and Bray–Curtis-based Raup-Crick Index (RCBray)

Null model analysis showed that the relative contributions of deterministic (|βNTI|≥ 2) and stochastic (|βNTI|≤ 2) processes in soil microbiota assembly were greatly affected by the host and the parasite, particularly for the bacterial microbiota (Fig. 3). For bacterial microbiota, the relative contribution of the stochastic process (BULK: 73.8%, NCD: 69.0%, RHA: 97.6%) was higher than the deterministic process (BULK: 26.2%, NCD: 31.0%, RHA: 12.4%) (Fig. 3 A). A higher relative contribution of the stochastic process mainly belonging to dispersal limitation was observed in the bacterial microbiota of RHA (77.1%) than in the bacterial microbiota of NCD (59.5%) and BULK (72.9%) (Fig. 3C). And the importance of heterogeneous selection (13.8%) and homogeneous selection (12.4%) was approximately equal in the BULK soil. However, the importance of homogeneous selection was higher in the bacterial microbiota of NCD (28.6%) than that in RHA (10.0%) (Fig. 3C). For fungal microbiota, only the stochastic process was in the microbiota of BULK, NCD and RHA (Fig. 3B). A higher relative contribution of the stochastic process mainly belonging to dispersal limitation was observed in the fungal microbiota of RHA (72.4%) than in the fungal microbiota of NCD (86.7%) and BULK (83.8%) (Fig. 3D). Collectively, the presence of the host increased the importance of homogeneous selection process and decreased the importance of dispersal limitation and heterogeneous selection processes in bacterial microbiota. And the presence of the parasitic plant increased the importance of dispersal limitation processes and decreased the importance of homogeneous selection processes.

### Diversity of rhizosphere and root microbiota of parasite and host under parasitism

**Fig. 4:**
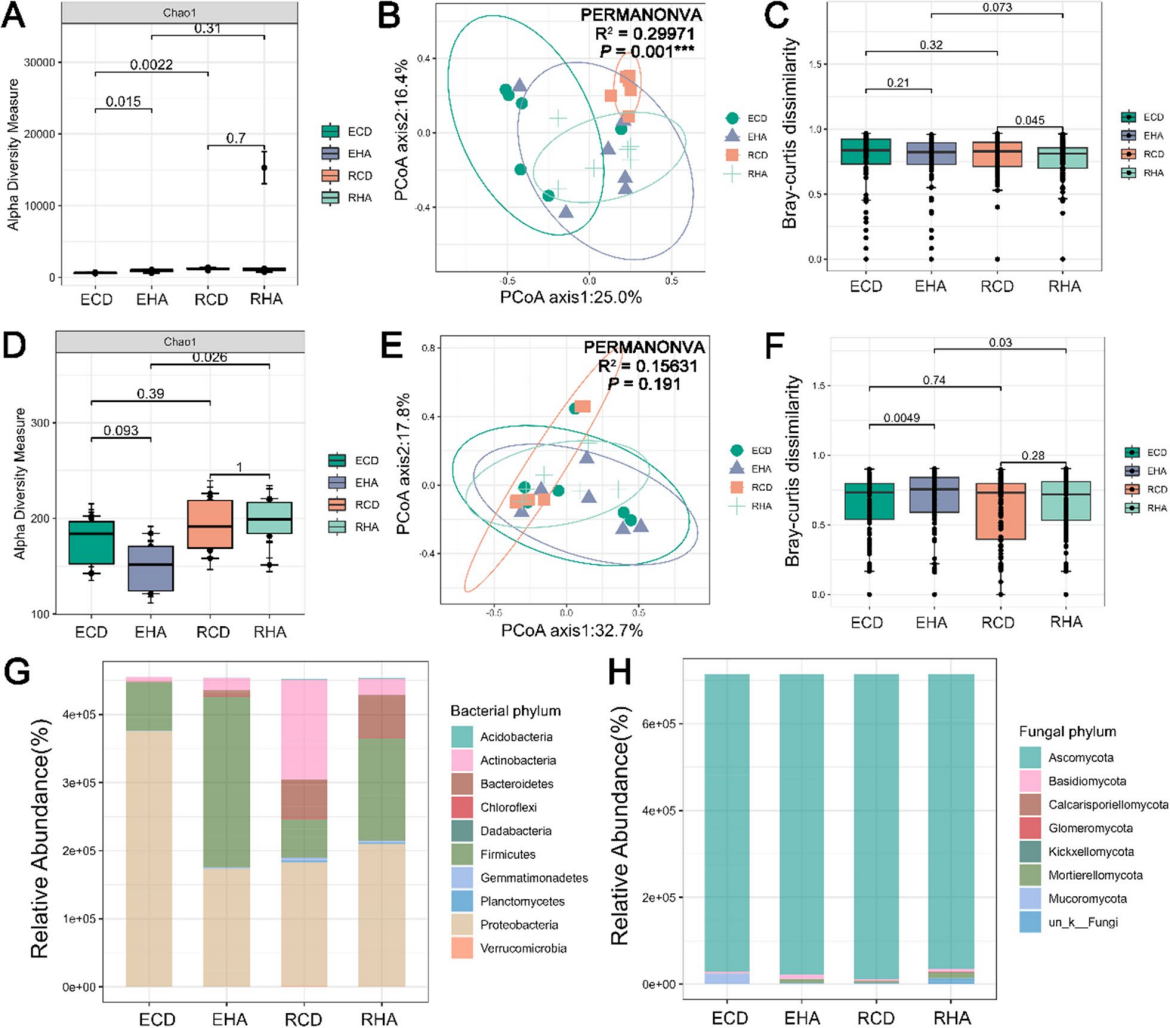
Diversity and distribution patterns of bacterial and fungal microbiota under parasitism. **A** and **D**. Alpha diversity of bacterial (**A**) and fungal (**D**) microbiota. The number on the horizontal line represented *P* value based on Wilcox test. **B** and **E** Principal-coordinate analysis (PCoA) ordinations based on Bray–Curtis distance matrices describing the distribution patterns of bacterial (**B**) and fungal (**E**) microbiota. The degree of variation explained (R^2^) and the significance (*P* values) provided by permutational multivariate analysis of variance (PERMANOVA) analysis. **C** and **F** Bacterial (**C**) and fungal (**F**) microbiota dissimilarity among four samples (RCD, RHA, ECD and EHA). The number on the horizontal line represented *P* value based on Wilcox test. **G** and **H**. The relative abundance of the major microbe phyla in bacterial (**G**) and fungal (**H**) microbiota

In both bacterial and fungal microbiota, the alpha diversity index was greater in the rhizosphere soil (RCD & RHA) than in the roots or stems (ECD and EHA) (Fig. 4; Additional file 1: Figs. S14, S15). For bacterial microbiota, the Shannon index showed that the rhizosphere bacterial microbiota of *H. ammodendron* (RHA) was significantly (*P* = 0.026) higher than that in the rhizosphere bacterial microbiota of *C. deserticola* (RCD) (Additional file 1: Fig. S14). However, other alpha indexes showed no significance (Fig. 4A; Additional file 1: Fig. S14). Observed, Chao1, ACE and Fisher index showed that the root bacterial microbiota of *H. ammodendron* (EHA) was significantly (*P* = 0.015) higher than that in stem bacterial microbiota of *C. deserticola* (ECD) (Additional file 1: Fig. S14). For fungal microbiota, alpha diversity between rhizosphere fungal microbiota of RHA and RCD had no significant difference (Fig. 4D; Additional file 1: Fig. S15). And Chao1 (*P* = 0.026) and ACE (*P* = 0.015) indexes showed that the rhizosphere fungal microbiota of RHA was significantly higher than the root fungal microbiota of EHA (Fig. 4D; Additional file 1: Fig. S15). PERMANOVA analysis and PCoA ordinations indicated that plant species explained the largest variation in both bacterial and fungal microbiota (bacteria, R^2^ = 0.29971, *P* = 0.001; fungi, R^2^ = 0.15631, *P* = 0.191) (Fig. 4 B and E; Additional file 1: Figs. S16, S17). Bacterial microbiota dissimilarity was significantly higher in the RCD than that in the RHA (*P* = 0.045) (Fig. 4C). In contrast to bacterial microbiota, the fungal microbiota dissimilarity between ECD and EHA (*P* = 0.0049) was significantly different (Fig. 4F). And fungal microbiota dissimilarity was significantly higher in the EHA than that in the RHA (*P* = 0.03) (Fig. 4F). *Proteobacteria*, *Actinobacteria*, *Firmicutes* and *Bacteroidetes* were the phyla with the highest abundance in the bacterial microbiota of *C. deserticola* and *H. ammodendron* (Fig. 4G; Additional file 1: Fig. S18 A), and *Ascomycota* was the phylum with the highest abundance in the fungal microbiota of *C. deserticola* and *H. ammodendron* (Fig. 4H; Additional file 1: Fig. S18B)*.*

For both bacterial and fungal microbiota, ternary plots were used to investigate the distribution of OTU within compartments. The distribution of OTU varied between the bacterial and fungal microbiota (Additional file 1: Fig. S19 A, B). OTU enriched and with high abundance were mainly found in the bacterial microbiota of the rhizosphere and plants (Additional file 1: Fig. S19A). Enrichment analysis showed that OTU1(3,5,6,9,54) were mainly enriched in the bacterial microbiota of root and stem (Additional file 1: Fig. S19A). More enriched OTU were found in the bacterial microbiota of *H. ammodendron* (Additional file 1: Fig. S19A). In contrast to the bacterial microbiota, enriched OTU in fungal microbiota were mainly concentrated in rhizosphere soil (Additional file 1: Fig. S19B). The enriched OTU were OTU1(3,5,6,9,54) in the fungal microbiota of the root and stem. And, more enriched fungal OTU were found in the fungal microbiota of *C. deserticola* (Additional file 1: Fig. S19B).

Within the bacteria, the enriched OTU included OTU1, which had 100% sequence identity to *Serratia rubidaea* [MT421936.1], OTU3, which had 100% sequence identity to *Pseudomonas sp.* [MZ505552.1], OTU5, which had 100% sequence identity to *Pantoea ananatis* [CP054912.1], OTU6, which had 100% sequence identity to *Bacillus atrophaeus* [MN826517.1], OTU9, which had 100% sequence identity to *Pantoea agglomerans* [MG733939.1] and OTU54, which had 100% sequence identity to *Erwinia sp.* [MF612164.1].

In contrast to the bacteria, the enriched OTU of the fungi included OTU1, which had 100% sequence identity to *Fusarium falciforme* [MT251175.1], OTU3, which had 100% sequence identity to *Botryotrichum piluliferum* [MH861633.1], OTU5, which had 100% sequence identity to *Chaetomium angustispirale* [MH864227.1], OTU6, which had 100% sequence identity to *Fusarium acuminatum* [MT566456.1], and OTU9, which had 100% sequence identity to *Monosporascus ibericus* [MK102683.1] (Additional file 2: Table S6).

### Co-occurrence network of rhizosphere and endosphere microbiota of parasite and host under parasitism

**Fig. 5:**
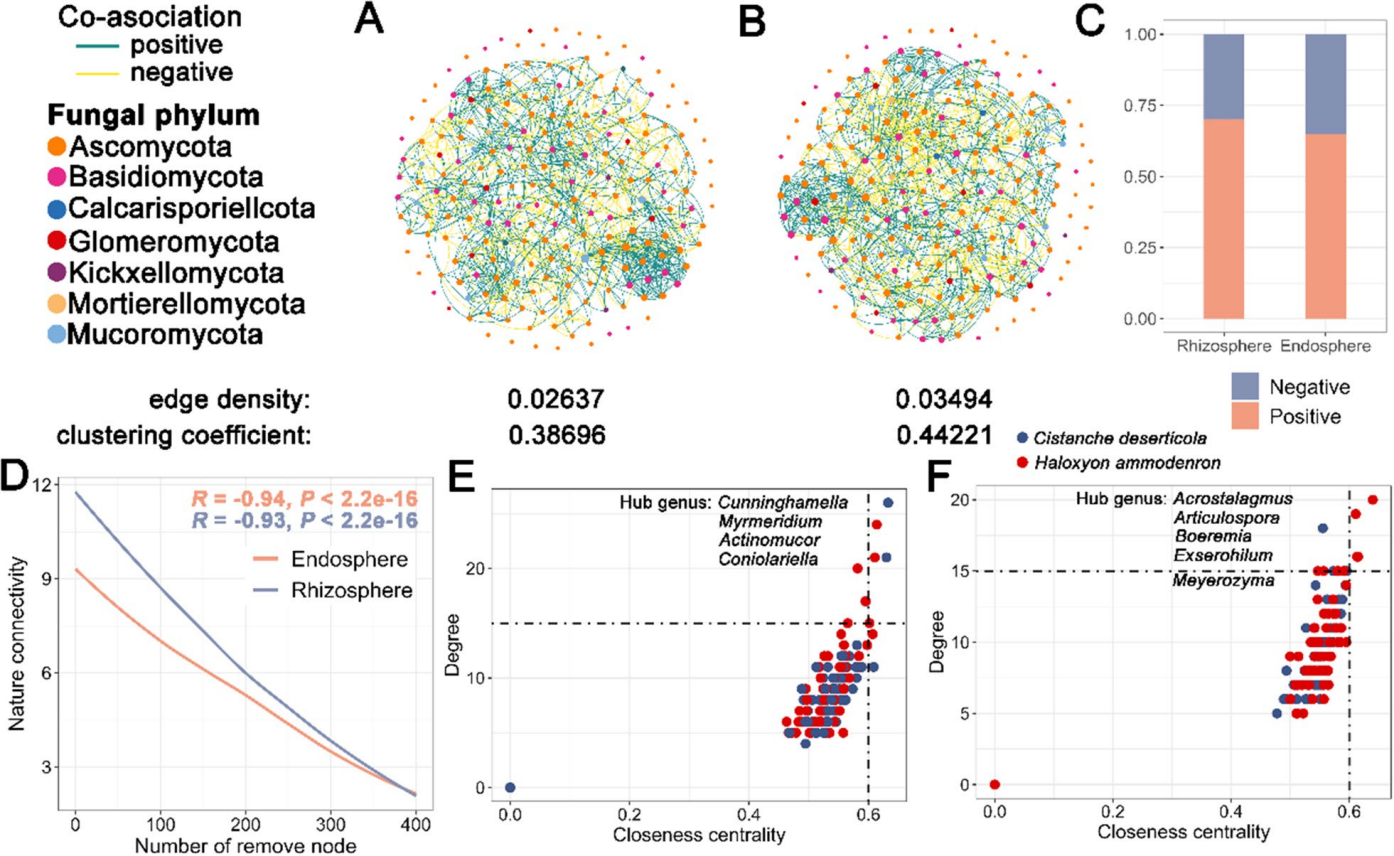
Complexity and stability of fungal microbiota network under parasitism. **A** and **B** Co-occurrence patterns of fungal microbiota networks in the rhizosphere (**A**) and endosphere (**B**) of *C. deserticola* and *H. ammodendron*. **C** The bar graph shows the proportion of positive and negative edges in fungal rhizosphere and endosphere microbiota network. **D** The robustness of the microbiota networks was based on natural connectivity. **E** and **F** Distribution patterns of the ‘hub nodes’ of fungal network in rhizosphere (**E**) and endosphere (**F**) microbiota network

To provide novel insight into co-occurrence patterns within the bacterial and fungal microbiota of *C. deserticola* and *H. ammodendron* under the parasitism status, microbial interaction networks for the rhizosphere and endosphere of *H. ammodendron* and *C. deserticola* were compared between bacteria and fungi (Fig. 5; Additional file 1: Figs. S20 and S21).

The proportion of the positive network edge (76.43%) was higher than the negative network edge (23.57%) at the rhizosphere bacterial network (Additional file 1: Fig. S20 B and Additional file 2: Table S7). Based on the natural connectivity analysis, the highest robustness was observed in the rhizosphere bacterial network (Additional file 1: Fig. S20 C). We further defined the ‘network hubs’ as nodes with high values of degree (> 40) and closeness centrality (> 0.6) in the bacterial network. We found 63 network hubs (*C. deserticola* 25, *H. ammodendron* 38) in the rhizosphere bacterial network, including *Baia*, *Tistlia*, *Microlunatus* genera and others (Additional file 1: Fig. S20 D and Additional file 2: Table S8).

**Fig. 6:**
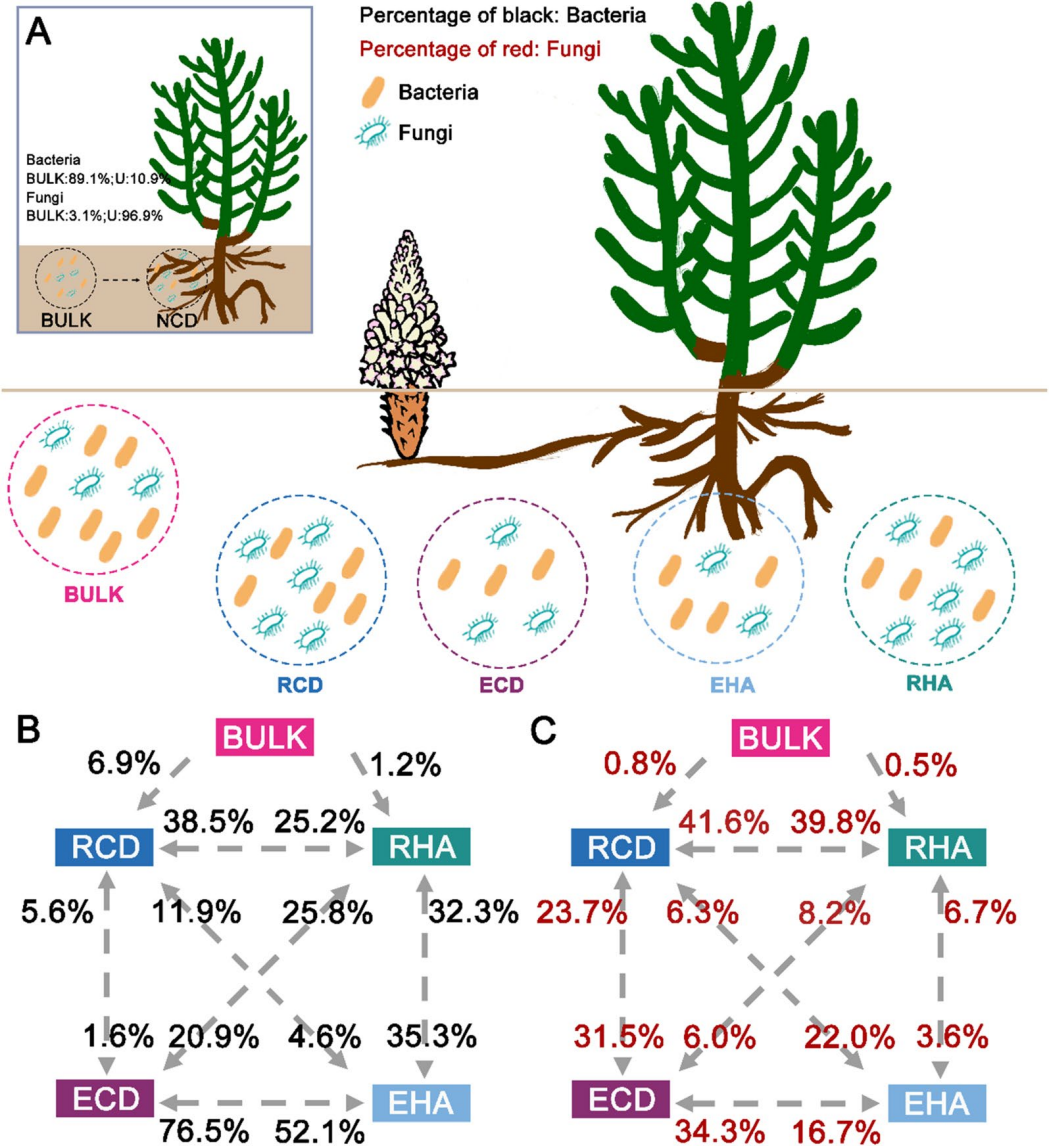
Source model of plant microbiome (SMPM) showing the potential sources of bacterial and fungal microbiota based on samples collected. Percentages in black were bacteria, and percentages in red were fungi. U, unknown source. **A** Potential sources of bacterial and fungal microbiota of NCD. **B** Potential sources of bacterial microbiota of *C. deserticola* and *H. ammodendron*. **C** Potential sources of fungal microbiota of *C. deserticola* and *H. ammodendron*

Edge density was greater in the endosphere (0.03494) fungal network than in the rhizosphere (0.02637) fungal network (Fig. 5A, B). However, modularity was greater in the rhizosphere (0.53264) fungal network (Additional file 2: Table S9), indicating that the connections within modules were greater than those between modules. Moreover, the proportion of the negative network edge markedly increased from 29.75% at the rhizosphere fungal network to 35.03% at the endosphere fungal network (Fig. 6C). Based on the natural connectivity analysis, the highest robustness was observed in the rhizosphere fungal network (Fig. 6D). We further defined the ‘network hubs’ as nodes with high values of degree (> 15) and closeness centrality (> 0.6) in the fungal network. We found 4 network hubs (*C. deserticola* 2, *H. ammodendron* 2) in the rhizosphere fungal network, including *Cunninghamella*, *Mymeridium*, *Actinomucor* and *Coniolariella* genera. And 5 network hubs (*C. deserticola* 1, *H. ammodendron* 4) were identified in the endosphere fungal network, including *Acrostalagmus*, *Articulospora*, *Boeremia*, *Exserohilum* and *Meyerozyma* genera (Fig. 6E, F).

### Community assembly mechanisms of rhizosphere and root microbiota of parasite and host under parasitism

We inferred community assembly mechanisms based on the null model and found that stochastic processes were the primary processes driving bacterial community assembly in both plant and rhizosphere microbiota (Fig. 3). The stochastic process was higher in rhizosphere bacterial microbiota (RHA: 87.6%, RCD: 95.2%) than in root or stem bacterial microbiota (EHA: 59.0%, ECD: 65.7%) (Fig. 3A). A higher relative contribution of deterministic processes mainly belonging to homogeneous selection was observed in bacterial microbiota of EHA (38.1%) and ECD (31.9%) than in bacterial microbiota of RHA (10.0%) and RCD (1.0%) (Fig. 3C). And only stochastic processes drove fungal community assembly in both plant and rhizosphere microbiota (Fig. 3B). A similar relative contribution of dispersal limitation was observed in the fungal microbiota of RHA, RCD, EHA and ECD (Fig. 3D).

### Potential sources of *C. deserticola* and *H. ammodendron* microbiota under parasitism

The Source Model of Plant Microbiome (SMPM) suggested that bacterial microbiota of NCD were mainly derived from bulk soils (Fig. 6A). However, the fungal microbiota of NCD was rarely derived from bulk soils (Fig. 6A).

Specifically, the endosphere bacterial microbiota of *C. deserticola* and *H. ammodendron* potentially selected the majority of taxa from a nearby species pool, with known source values > 96%. The main potential sources of the endosphere bacterial microbiota of the parasite were the endosphere bacterial microbiota of the host (76.5%), and the second potential sources of the bacterial microbiota of the parasite were the rhizosphere bacterial microbiota of the parasite (20.9%) (Fig. 6B). The endosphere bacterial microbiota of the parasite (52.1%) were the main potential sources of the endosphere bacterial microbiota of the host (Fig. 6B). And the rhizosphere bacterial microbiota of the host (35.3%) was the second potential source of the endosphere bacterial microbiota of the host (Fig. 6B). However, unknown sources accounted for 15.4–37.1% of the potential sources of rhizosphere bacterial microbiota, indicating that other environmental sources might have contributed to rhizosphere bacterial microbiota. And the bacterial microbiota of bulk soil contributed little to the potential sources of rhizosphere bacterial microbiota. For the rhizosphere bacterial microbiota of *C. deserticola*, the rhizosphere and endosphere bacterial microbiota of *H. ammodendron* accounted 38.5% and 11.9% for the potential source (Fig. 6B). Relative to the rhizosphere bacterial microbiota of *C. deserticola*, the main potential sources of rhizosphere bacterial microbiota of *H. ammodendron* were the endosphere bacterial microbiota of the parasite (25.8%) and the host (32.3%), and the rhizosphere bacterial microbiota of *C. deserticola* (25.2%) (Fig. 6B).

For fungal microbiota, unknown sources accounted for 27.7%-52.5% of the potential sources of fungal microbiota, indicating that other environmental sources might have contributed to fungal microbiota. The main potential source of the rhizosphere fungal microbiota of *H. ammodendron* was the rhizosphere fungal microbiota of *C. deserticola* (39.8%) (Fig. 6C). For the rhizosphere fungal microbiota of *C. derserticola*, the rhizosphere fungal microbiota of *H. ammodendron* accounted 41.6% for the potential sources, and the endosphere fungal microbiota of *C. deserticola* ranked the second (23.7%) (Fig. 6C). Relative to rhizosphere fungal microbiota, the endosphere fungal microbiota of the parasite (or the host) and the rhizosphere fungal microbiota of *C. deserticola* were the main potential sources of endosphere fungal microbiota.

## Discussion

In this study, we explored the diversity, composition, co-occurrence network structures and community assembly mechanisms of the rhizosphere bacterial and fungal microbiota of *H. ammodendron* from not parasitized to parasitized. Also, we described the rhizosphere and endosphere bacterial and fungal microbiota of *C. deserticola* and *H. ammodendron* under parasitism for the first time.

The parasitic plant has an influence on the diversity of bacterial and fungal microbiota, which is consistent with previous work reported by Schmidt [47], who demonstrated that host selection processes moderated the influence of agricultural management on rhizosphere microbial communities. The following reasons are as offered for the possible mechanism: modification of rhizosphere soil is conducted by plants through moisture, oxygen and nutrient uptake from the rhizosphere, rhizodeposition and production of root exudates. Hence, the diversity of the rhizosphere microbiota of plants could be changed. An increasing number of studies have been conducted to prove that plants select specific rhizosphere bacteria and fungi actively to build a suitable habitat favorable for themselves [4, 48, 49]. One way plants influence soil properties is to select a subset of bulk soil bacterial populations with the genetic and metabolic traits to subsist and grow in the rhizosphere. In turn, it results in the reduced diversity of microbiota that characterizes the rhizosphere effect [7, 50].

To evaluate the complexity of targeted microbiota, co-occurrence network analysis was used [51, 52]. Various investigations indicated that the rhizosphere effect screens bacterial microbiota, influences their assemblages, and promotes more complex network dynamics in the rhizosphere compared with bulk soil [8, 53, 54]. In addition, many studies have shown that the network structure of fungi is much simpler than that of bacteria under the same circumstances. And plants also contribute to the complexity of plant fungal networks [14, 47, 55]. Our study demonstrated that the host *H. ammodendron* increased the nodes, links and modules of the fungal network when compared with the bulk soil. However, the parasite *C. deserticola* decreased the complexity of the co-occurrence network, including the number of nodes, links and modules. The concept of ‘parasitic reduction syndrome’ was extended to include a reduction in co-occurrence network structure, which was the same as *Orobanche* and *Heder* [43]. Interestingly, the bacterial and fungal network structure of BULK and RHA was highly similar. We speculated that the possible reason why the presence of *C. deserticola* shaped the network structure of the rhizosphere microbiota of *H. ammodendron* was the release of root exudates from *H. ammodendron* when parasitism formed. Strigolactones, a kind of carotenoid-derived molecule, plays an important role in the seed germination of parasitic plants and the formation of parasitism [56, 57]. Moreover, strigolactones are a bridge that links rhizobacterial swarming and nodule initiation in legumes and interactions with oomycete pathogens, bacteria and fungi [58, 59]. Meanwhile, the inference that stochastic processes were the primary processes driving bacterial and fungal community assembly in both plant and rhizosphere microbiota also provided evidence that root exudates might play an important role in microbiota assemblages. The three-phase conceptual model showed that the initial establishment of microbial communities is expected to be dominated by stochasticity [60]. In our study, the harvesting of *C. deserticola* and *H. ammodendron* was a vegetative growth period, which is consistent with the idea that stochasticity is likely to dominate the initial phase of community assembly. In a way, the definition of the initial phase of community assembly was when a broad range of organisms can grow successfully in a given environment [61, 62]. And it had also been suggested that the sugars released by seedling roots in soil provided a resource rich environment that reduces competitive pressures, which led to a dominance of stochasticity during the initial establishment of rhizosphere communities [63–65]. However, whether the strigolactones or root exudates could influence the microbiota of the parasite and the host is still unknown and requires in-depth study utilizing a scope of parasitic and host plants.

Module hubs (also called hub nodes) are network nodes, in which removal may cause the disassembly of modules or networks [66, 67]. And hub nodes represent keystone species in an ecosystem [68, 69]. In the co-occurrence network of fungal microbiota, 407 and 416 connections between *C. deserticola* and *H. ammodendron* were identified in the rhizosphere and endosphere networks separately, indicating that the numerous fungi communicated between parasite and host. Interestingly, four hub nodes of *H. ammodendron* in the endosphere microbiota were identified, while one hub node of *C. deserticola* in the endosphere microbiota was identified. Similarly, more hub nodes were found in *H. ammodendron* in the rhizosphere bacterial co-occurrence network. It indicated that *H. ammodendron* played a predominant role in the co-occurrence network of endosphere fungal and rhizosphere bacterial microbiota. We suspected that it might be due to the non-photosynthetic characteristics of *C. deserticola*. *C. deserticola* is a holoparasite lacking photosynthetic functions, completely dependent on the host *H. ammodendron* for water and nutrients. Hence, *C. deserticola* may also utilize the endosphere fungal microbe which transfers from the host through haustoria. In the process of evolution, *C. deserticola* formed a unique survival and development strategy. To date, very few studies have examined hub node genera for bacteria and fungi in a stable parasitism state. Fitzpatrick reported that the relative abundance of *Burkholderiales* was strongly correlated with the bacterial microbiota of *Orobanche* and *Hedera* [43]. It supported that the *Burkholderiales* were a strong contributor to the congruence between root communities of parasites and hosts [43]. Here, we identified sixty-three bacterial and nine fungal genera strongly correlated to the bacterial and fungal microbiota of *C. deserticola* and *H. ammodendron*.

Haustorial connections are likely to allow the flow or even exchange of various molecules between parasites and hosts, including mRNA, small RNA, protein and phytoplasmas [70–72]. However, whether and to what extent microbes transfer between parasites and hosts remains poorly studied. In this study, SMPM suggested that the endosphere microbiota of *H. ammodendron* were the main potential sources of the endosphere microbiota of *C. deserticola*. Similarly, the potential sources of the endosphere microbiota of *H. ammodendron* included the endosphere microbiota of *C. deserticola*. However, the potential source percentage from parasites to hosts was lower than the potential source percentage from hosts to parasites, illustrating that microbial communication was bidirectional but mainly from hosts to the parasites. Moreover, it revealed that parasitic plants relied on their host via the haustorium not only for nutrients, energy and water, but also for microbes. Meanwhile, the parasite also transmitted the microbes to hosts as a signal for cooperative adaptation.

## Conclusions

Here, we present, for the first time, a comprehensive exploration of bacterial and fungal dynamics and community assembly in the rhizosphere of a host (*H. ammodendron*) as its roots were parasitized by a parasite (*C. deserticola*). Furthermore, we revealed the steady-state parasitic spectrum of the bacterial and fungal microbiota of *C. deserticola* and *H. ammodendron*. Our results answered the three scientific questions posed in the introduction. First, parasites reduced the diversity, simplified the composition and co-occurrence network structure and increased the proportion of stochastic processes mainly belonging to dispersal limitation in bacterial microbiota of *H. ammodendron*. Second, high similarities were found in diversity, composition and percentage of stochastic processes in community assembly mechanisms between rhizosphere and endosphere microbiota of *C. deserticola* and *H. ammodendron*. And *H. ammodendron* played a predominant role in the co-occurrence network of rhizosphere and endosphere microbiota. Third, the potential source percentage from parasites to host was lower than the potential source percentage from hosts to parasites, illustrating that microbial communication was bidirectional but mainly from hosts to the parasites. We assumed that *C. deserticola* and *H. ammodendron* formed a shared microbial community in an arid ecosystem. Understanding the bacterial and fungal microbiota patterns of *C. deserticola* and *H. ammodendron* is fundamental to understanding the microbiota landscape of the desert ecosystem containing parasitic plants and their hosts.

## Supplementary Information

**Additional file 1.** Supplementary figures.

**Additional file 2.** Supplementary tables.

## Data Availability

The raw sequence data reported in this paper are available in the NCBI Sequence Read Archive under BioProject PRJNA761515 and PRJNA761516.

## Abbreviations

BULK: Bulk soil
NCD: Rhizosphere soil of *H. ammodendron* non-parasited
RHA: Rhizosphere soil of *H. ammodendron* parasited
RCD: Rhizosphere soil of *C.deserticola*
EHA: *Haloxylon ammodendron* Root
ECD: *Cistanche deserticola* Stem
