# Supplementary material for: From guest to host: parasite Cistanche deserticola shapes and dominates bacterial and fungal community structure and network complexity
Source: Environ Microbiome. 2023 Feb 22;18:11. doi: 10.1186/s40793-023-00471-3 (PMC9945605; doi:10.1186/s40793-023-00471-3)
Supplement: Supplementary file 1 — Additional file 1. Supplementary figures. [file 40793_2023_471_MOESM1_ESM.docx]

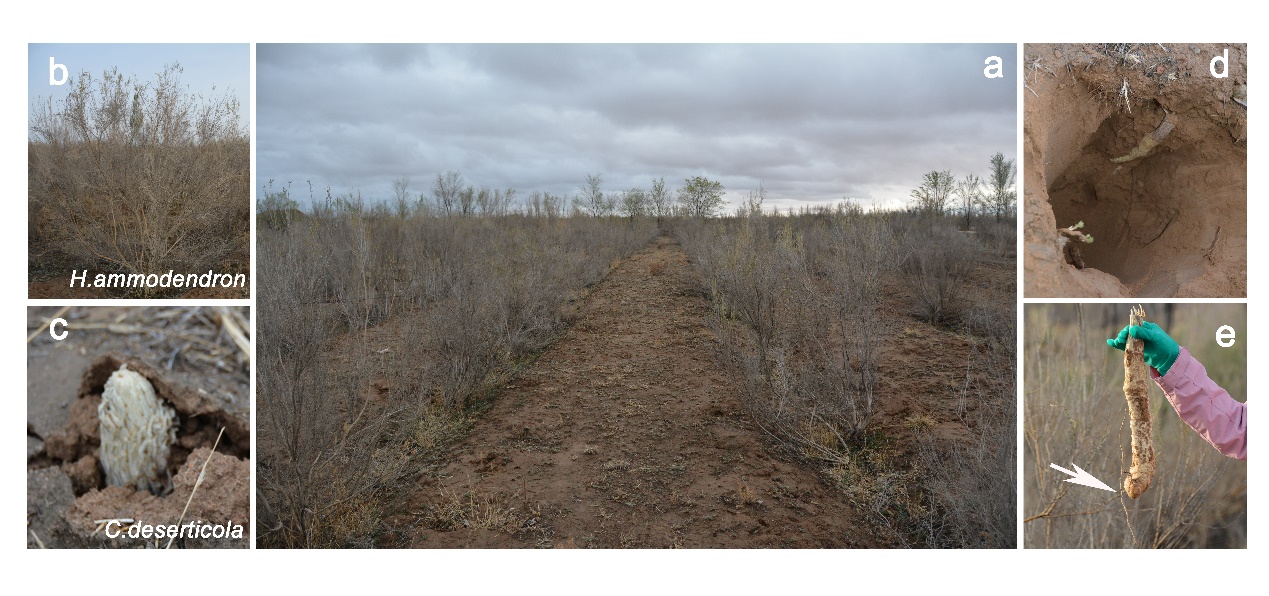


Figure S1. Habitat niches and rhizosphere-root systems of *H. ammodendron* and *C. deserticola*. A) Photograph of the sampling point - ‘Chinese Cistanche planting’ base. B) Photograph of *H. ammodendron* plant. C) Photograph of *C. deserticola* plant. D) Photograph of the underground part of *C. deserticola*. E) Stem of *C. deserticola* plant and root of *H. ammodendron* plant.


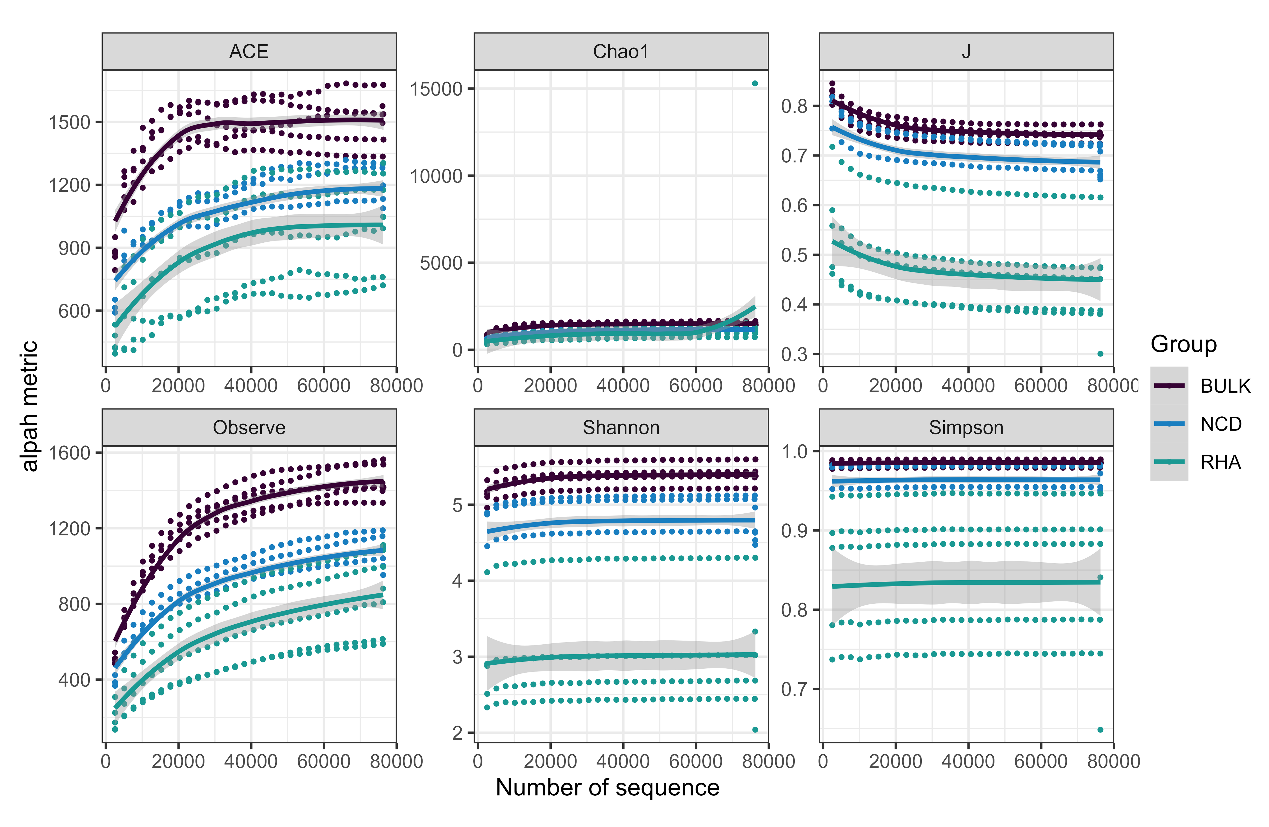


Figure S2 Mean rarefaction curves for bacterial microbiota of BULK, NCD and RHA. Rarefaction curves were determined for ACE, Chao1, J, Observed species, Shannon and Simpson index.


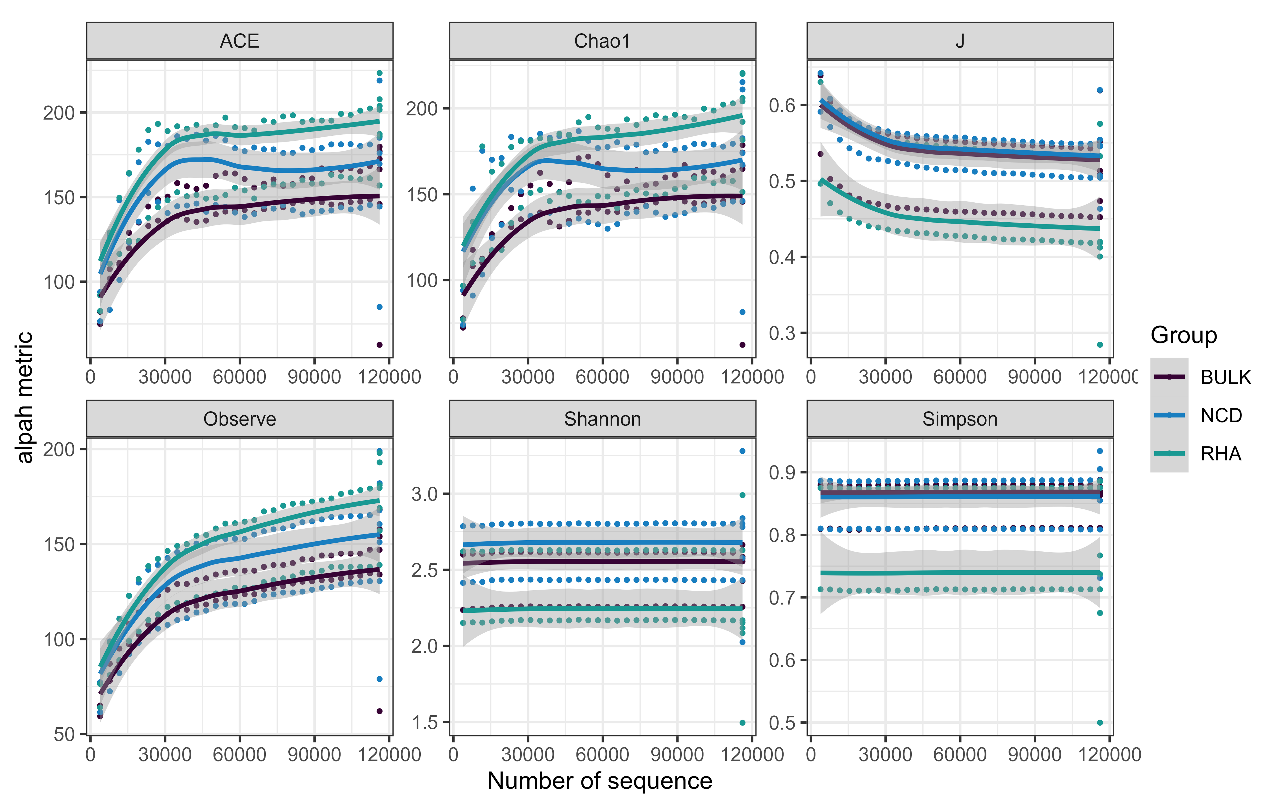


Figure S3 Mean rarefaction curves for fungal microbiota of BULK, NCD and RHA. Rarefaction curves were determined for ACE, Chao1, J, Observed species, Shannon and Simpson index.


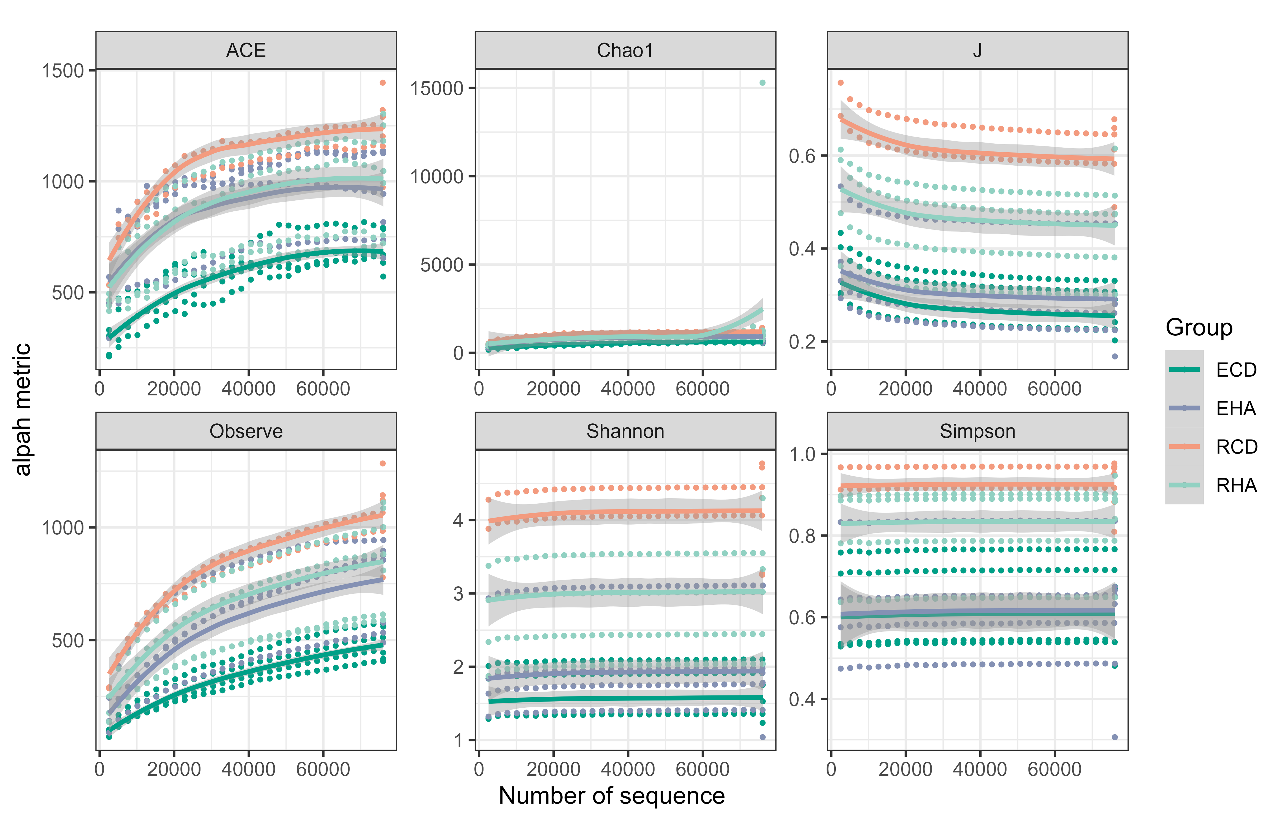


Figure S4 Mean rarefaction curves for bacterial microbiota of ECD, EHA, RCD and RHA. Rarefaction curves were determined for ACE, Chao1, J, Observed species, Shannon and Simpson index.


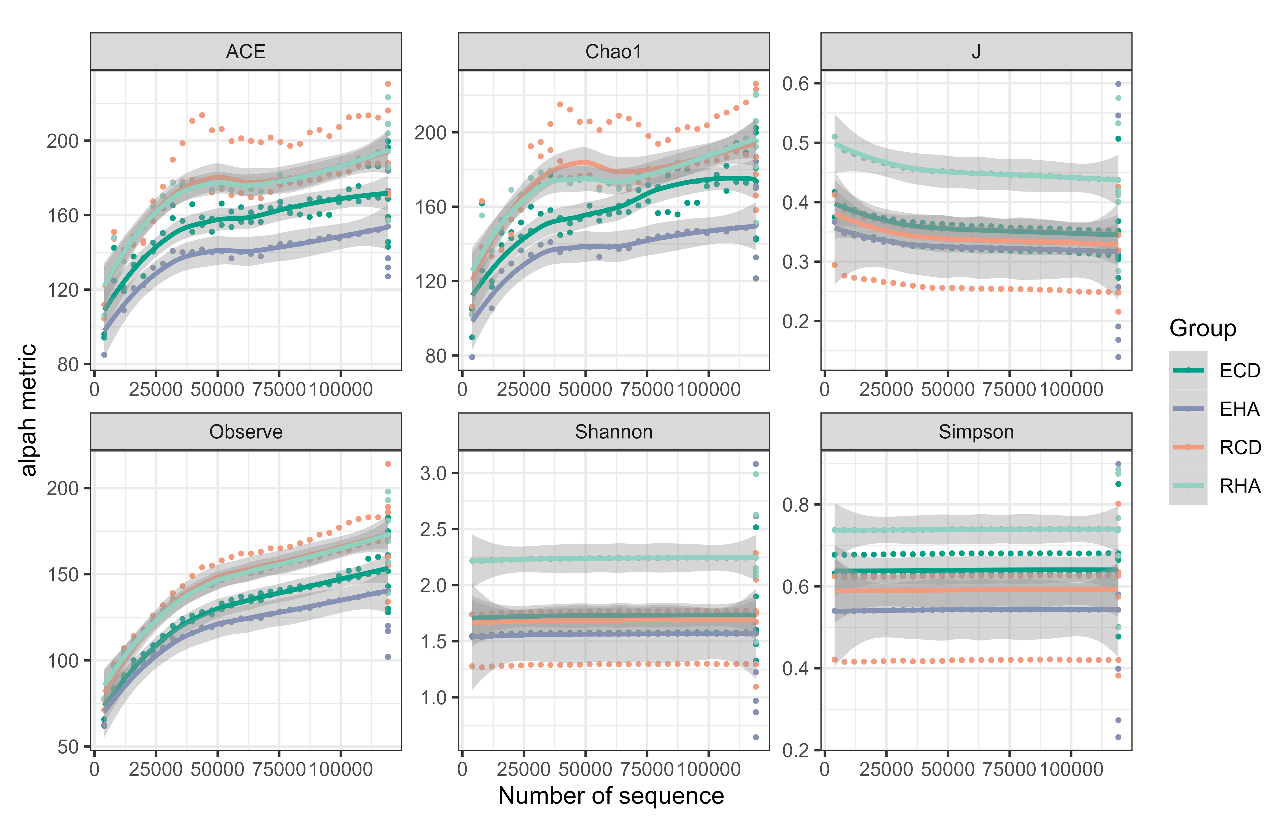


Figure S5 Mean rarefaction curves for fungal microbiota of ECD, EHA, RCD and RHA. Rarefaction curves were determined for ACE, Chao1, J, Observed species, Shannon and Simpson index.


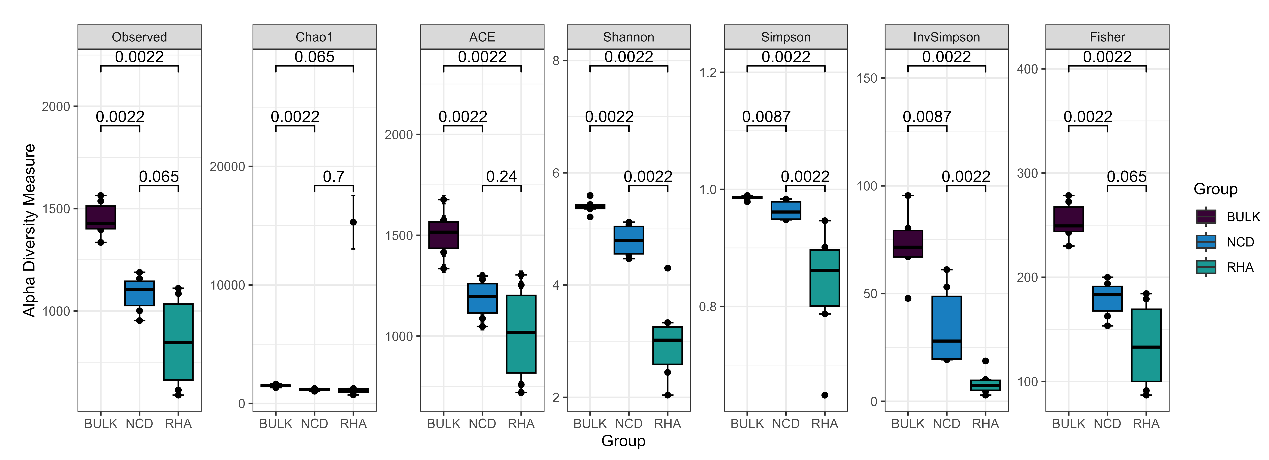


Figure S6 Measures of bacterial α-diversity across BULK, NCD and RHA samples. Observed richness, Chao1, ACE, Shannon, Simpson, InvSimpson and Fisher index.


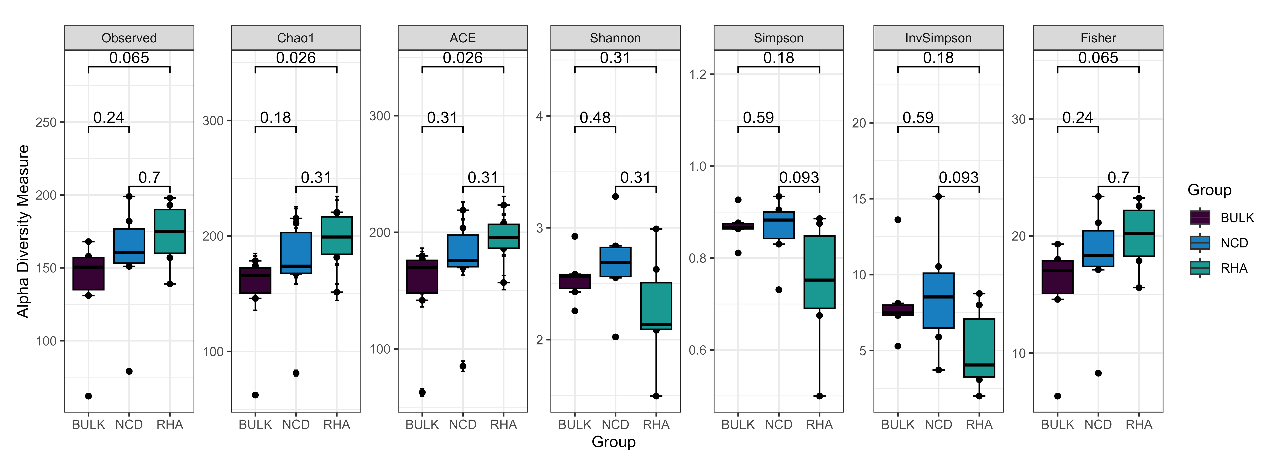


Figure S7 Measures of fungal α-diversity across BULK, NCD and RHA samples. Observed richness, Chao1, ACE, Shannon, Simpson, InvSimpson and Fisher index.


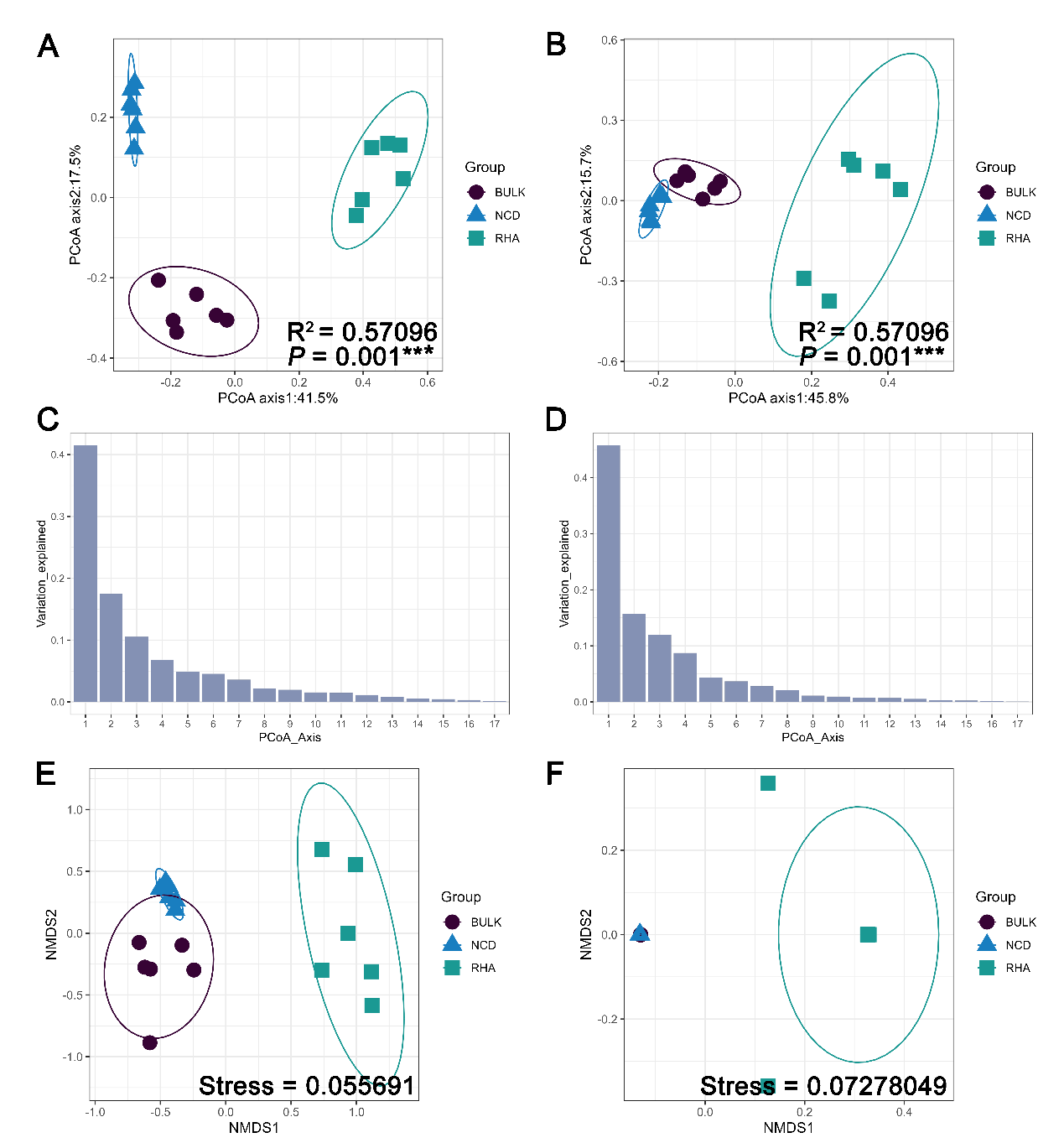


Figure S8 Principle coordinate analyses, corresponding scree plots and non-metric multidimensional scaling analyses associated with different measures of bacterial microbiota dissimilarity. (A and C) Bray-Curtis dissimilarity. (B and D) UniFrac dissimilarity. E Bray-Curtis dissimilarity. F UniFrac dissimilarity.


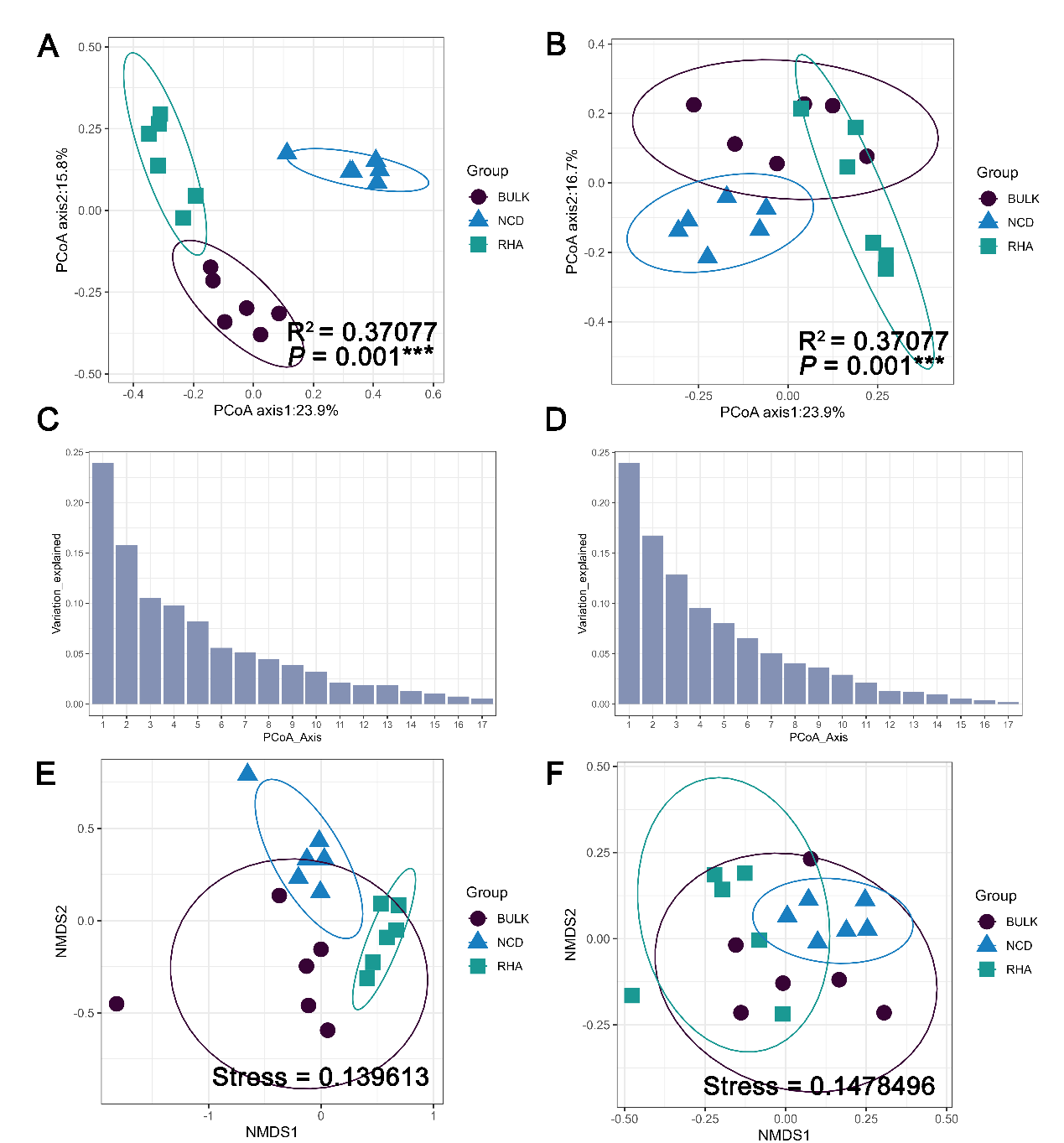


Figure S9 Principle coordinate analyses, corresponding scree plots and non-metric multidimensional scaling analyses associated with different measures of fungal microbiota dissimilarity. (A and C) Bray-Curtis dissimilarity. (B and D) UniFrac dissimilarity. E Bray-Curtis dissimilarity. F UniFrac dissimilarity.


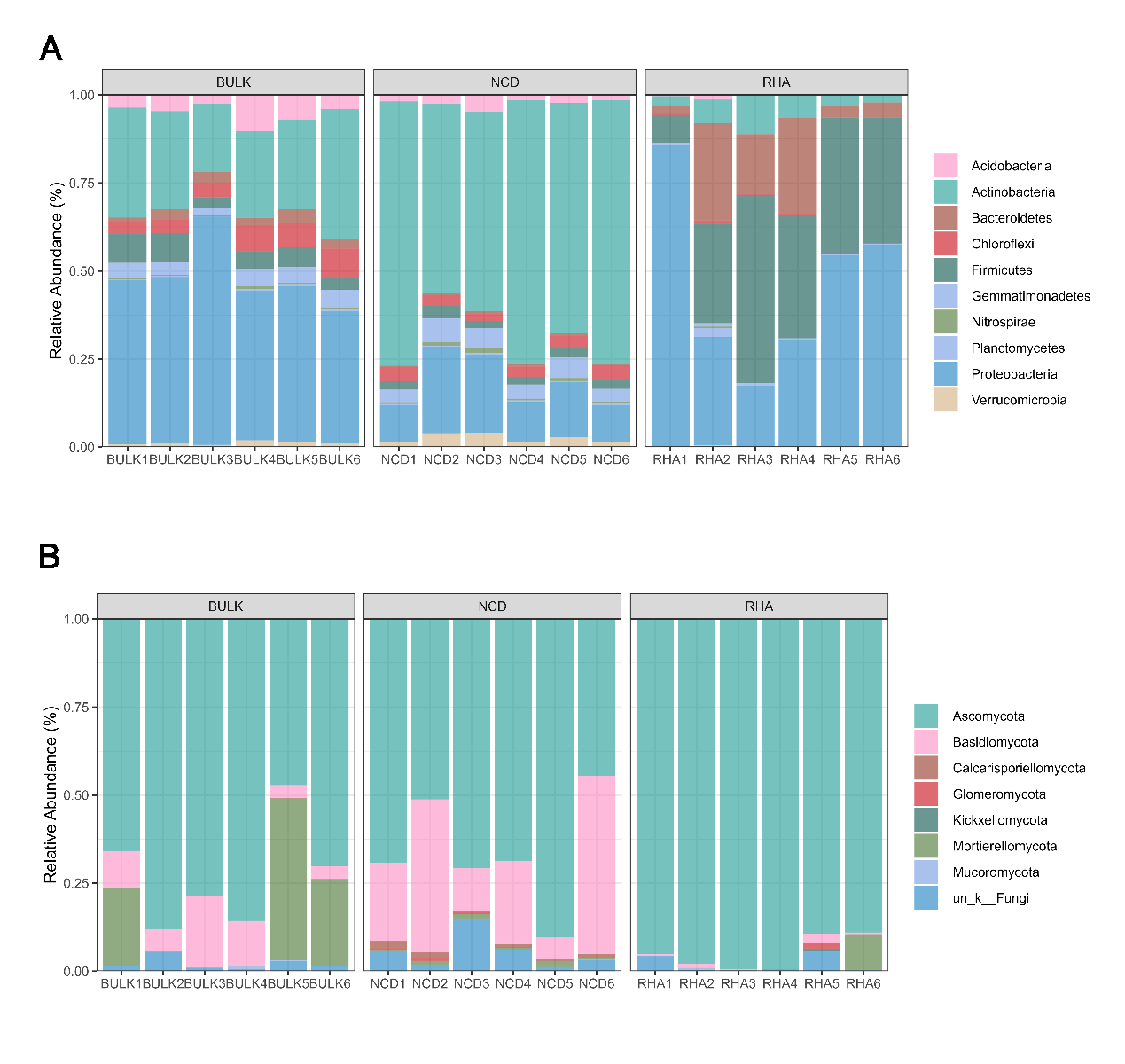


Figure S10 Relative abundance of the major bacterial and fungal microbiota found in BULK, NCD and RHA. A. Bacteria. B. Fungi.


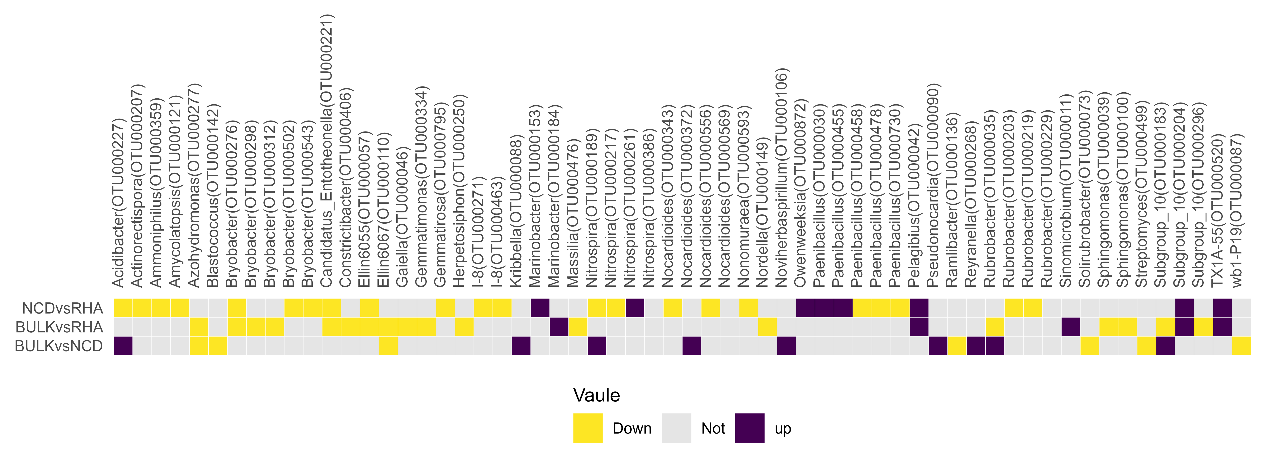


Figure S11 Differential abundance patterns of bacteria in BULK, NCD and RHA using ALDEx2. Down: significantly down expressed OTU identified by Deseq2; Up: significantly up expressed OTU identified by Deseq2.


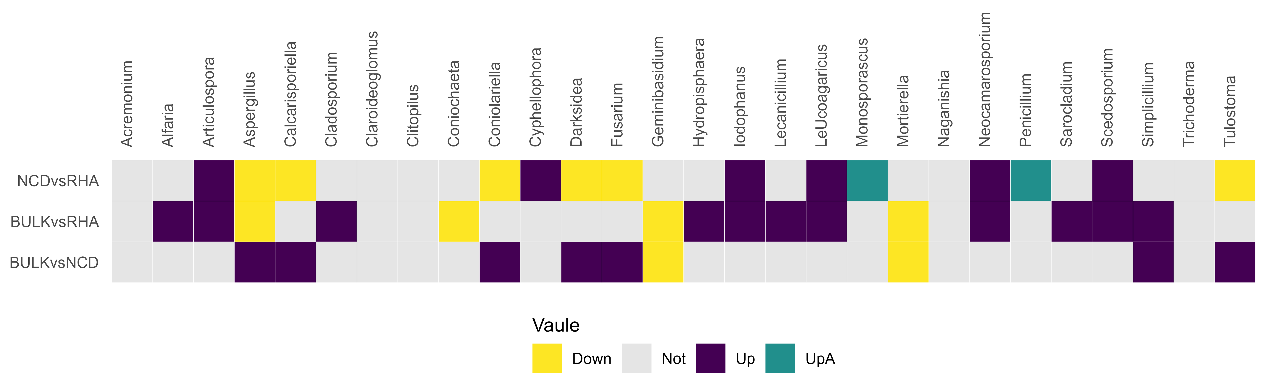


Figure S12 Differential abundance patterns of fungal in BULK, NCD and RHA using ALDEx2 and Deseq2. Down: significantly down expressed OTU identified by Deseq2; Up: significantly up expressed OTU identified by Deseq2; UpA: significantly up expressed OTU identified by ALDEx2.


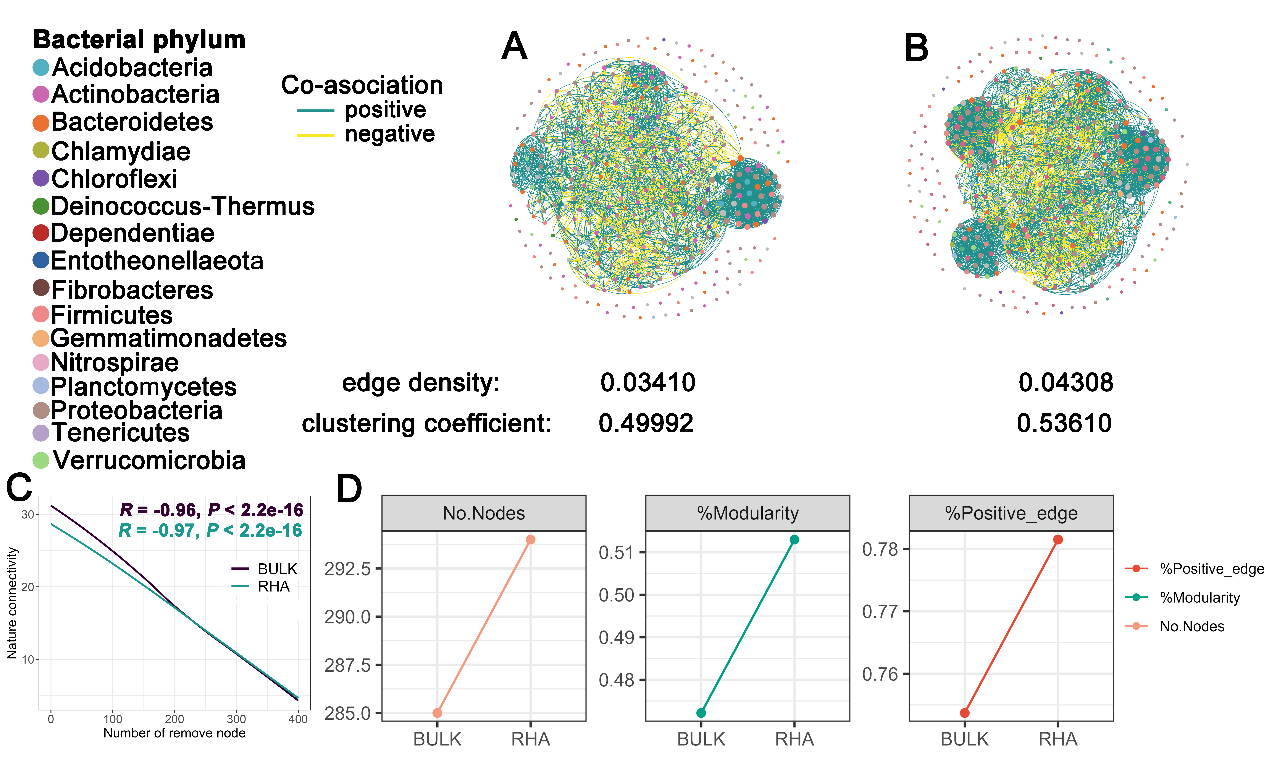


Figure S13 Bacterial microbiota networks for BULK and RHA. A and B. Co-occurrence patterns of bacrerial microbiota networks in BULK (A) and RHA (B). C. The robustness of microbiota networks was based on natural connectivity. D. Network topological parameters for bacterial microbiota networks.


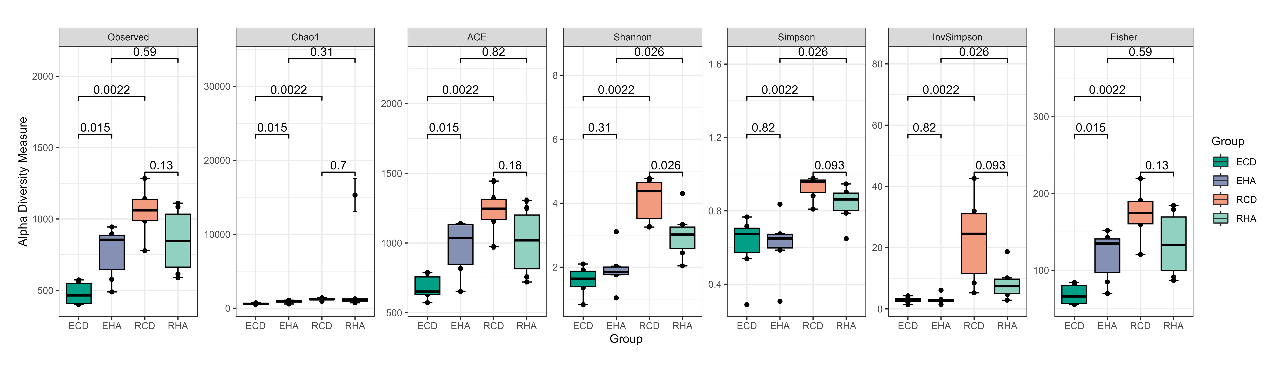


Figure S14 Measures of bacterial α-diversity across ECD, EHA, RCD and RHA samples. Observed richness, Chao1, ACE, Shannon, Simpson, InvSimpson and Fisher index.


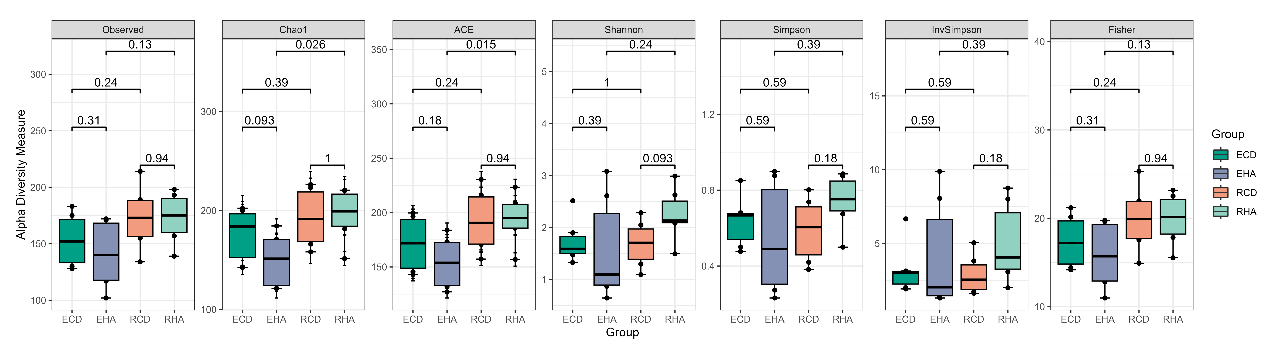


Figure S15 Measures of fungal α-diversity across ECD, EHA, RCD and RHA samples. Observed richness, Chao1, ACE, Shannon, Simpson, InvSimpson and Fisher index.


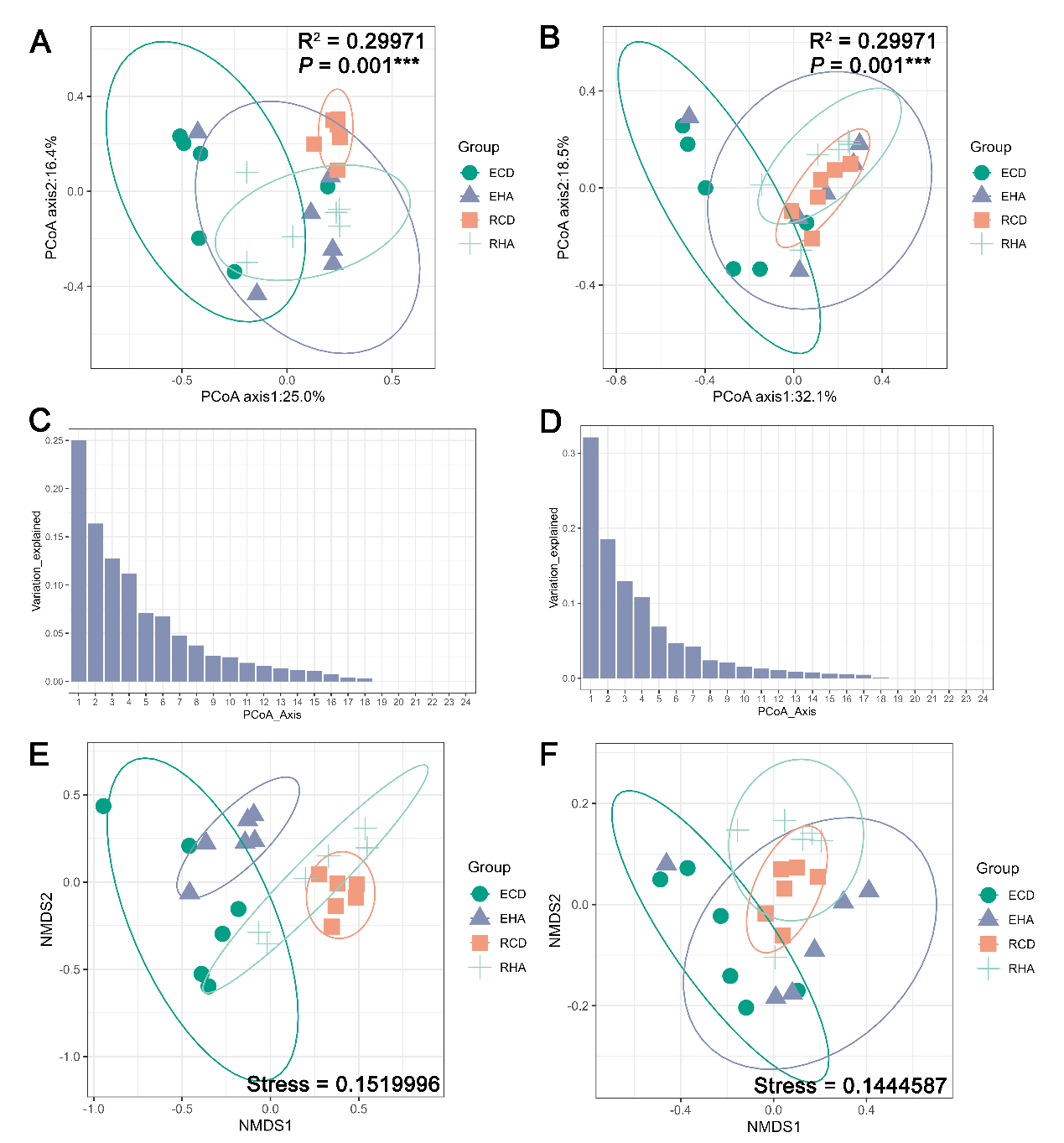


Figure S16 Principle coordinate analyses, corresponding scree plots and non-metric multidimensional scaling analyses associated with different measures of bacterial microbiota dissimilarity. (A and C) Bray-Curtis dissimilarity. (B and D) UniFrac dissimilarity. E Bray-Curtis dissimilarity. F UniFrac dissimilarity.


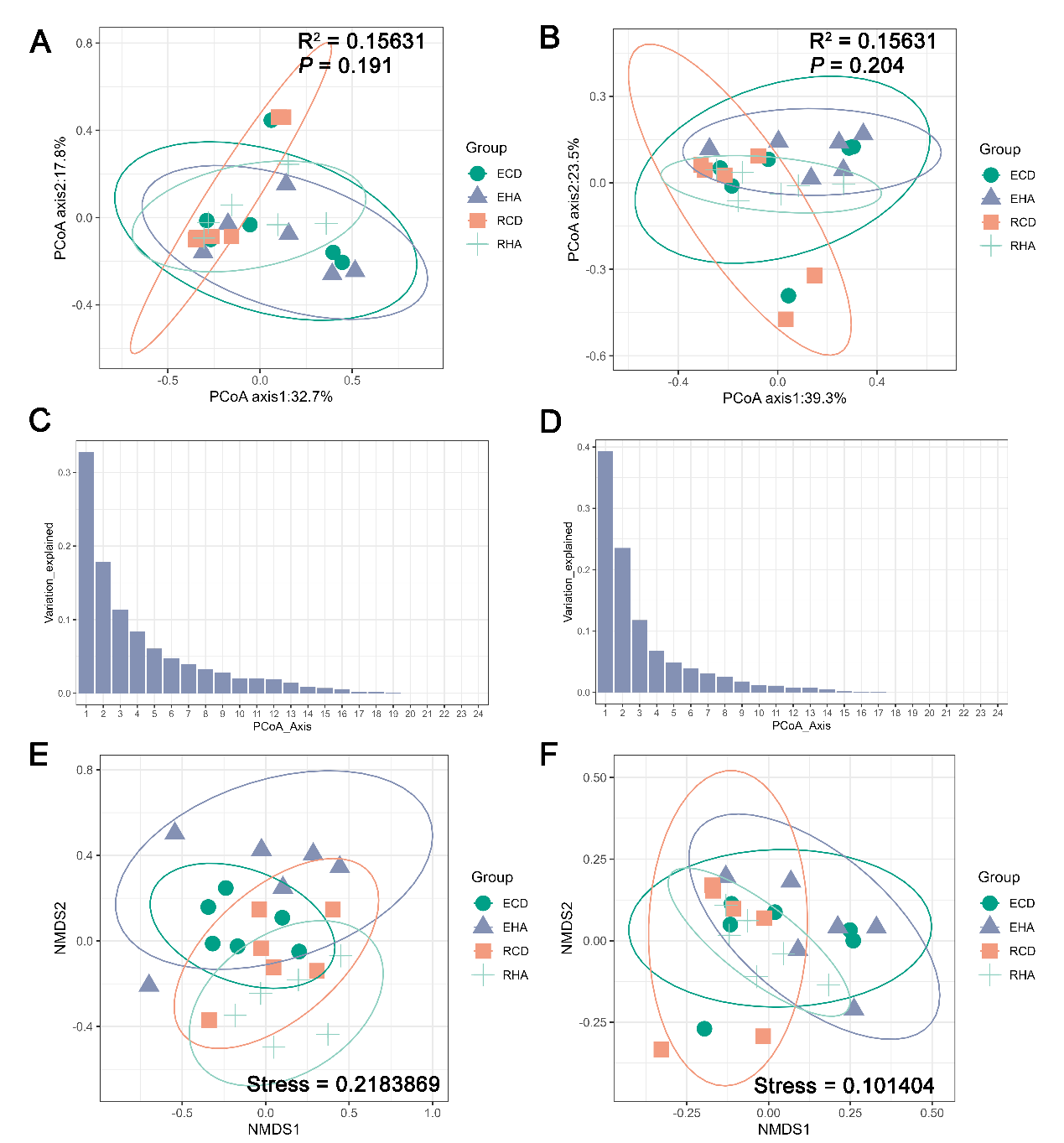


Figure S17 Principle coordinate analyses, corresponding scree plots and non-metric multidimensional scaling analyses associated with different measures of fungal microbiota dissimilarity. (A and C) Bray-Curtis dissimilarity. (B and D) UniFrac dissimilarity. E Bray-Curtis dissimilarity. F UniFrac dissimilarity.


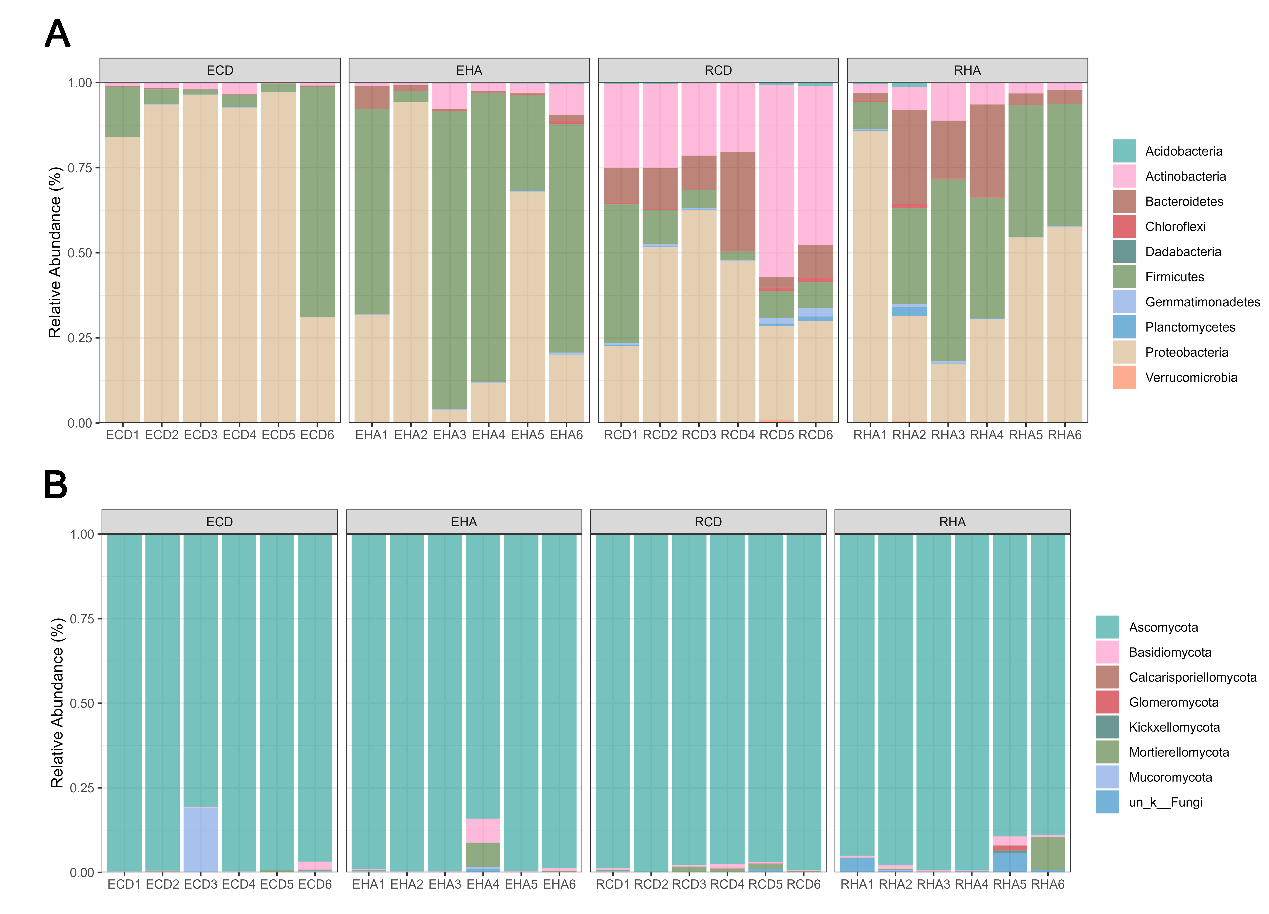


Figure S18 Relative abundance of the major bacterial and fungal microbiota found in ECD, EHA, RCD and RHA. A. Bacteria. B. Fungi.


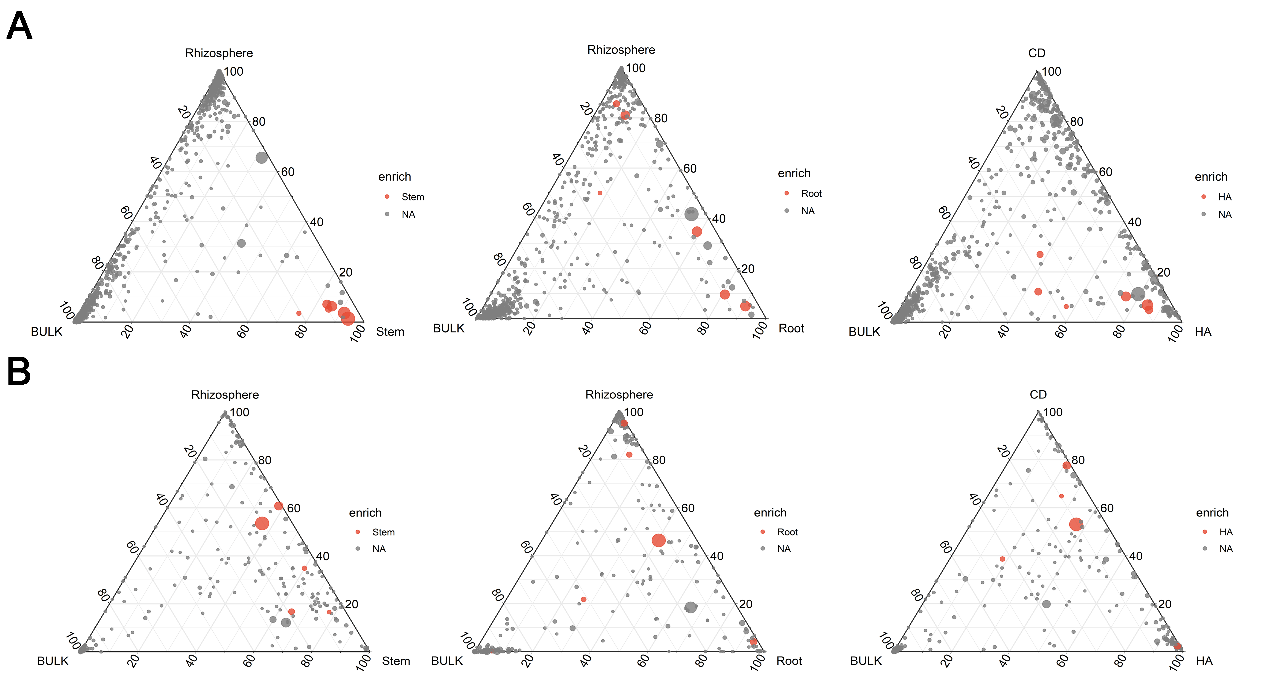


Figure S19 Ternary plots of OTU distribution among bacterial (A) and fungal (B) microbiota of bulk soil, rhizosphere soil and stem (root). Red dots represented the enriched OTU whose *P* value was less than 0.05 after Kruskal-Wallis test, and grey dots represented not enriched OTU whose *P* value was greater than 0.05 after Kruskal-Wallis test. The size of the dots represented the average relative abundance of OTU. CD-*Cistanche deserticola*; HA-*Haloxylon ammodendron*.


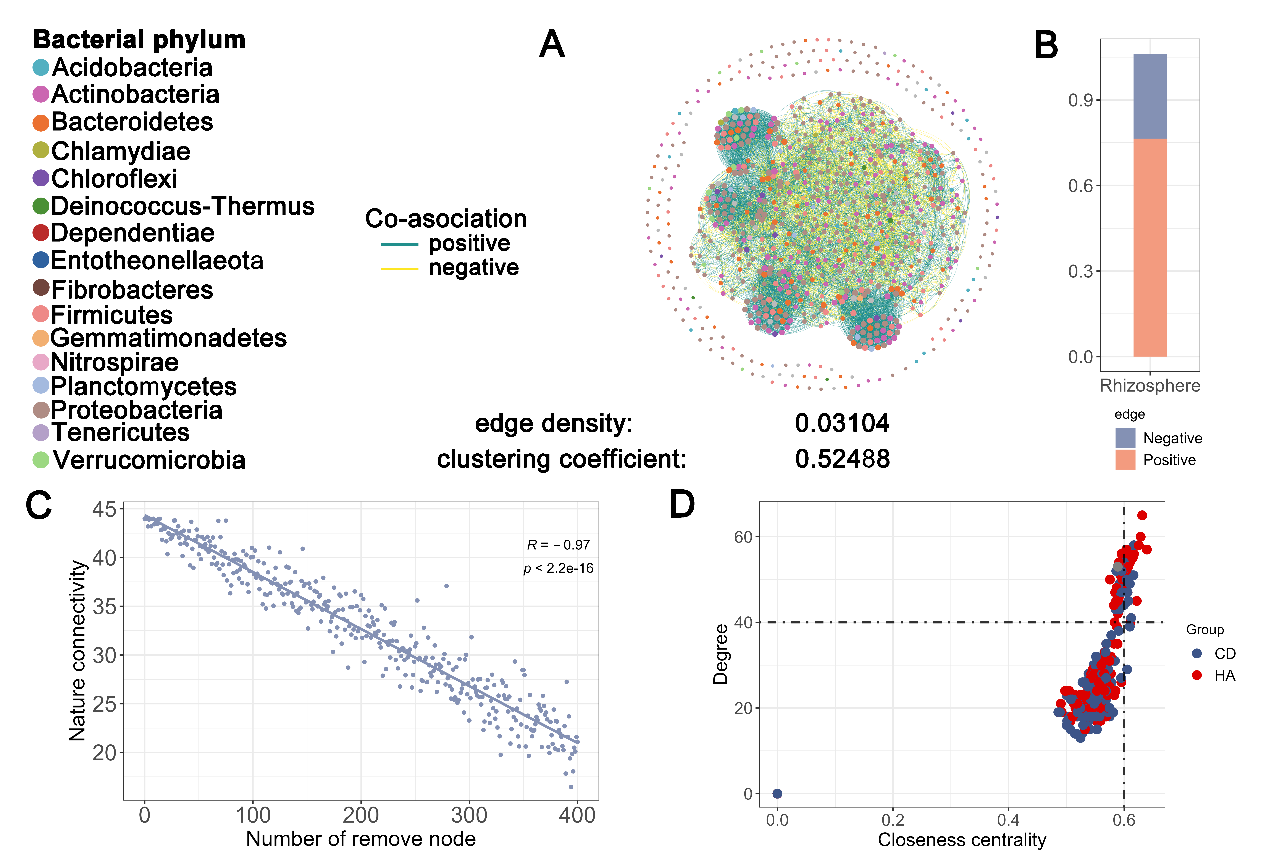


Figure S20 Complexity and stability of rhizosphere bacterial microbiota network under parasitism. A. Co-occurrence patterns of bacterial microbiota networks in the rhizosphere. B. The bar graph shows the proportion of positive and negative edges in rhizosphere microbiota network. C. The robustness of the microbiota networks was based on natural connectivity. D. Distribution patterns of the ‘hub nodes’ of fungal network in rhizosphere microbiota network.


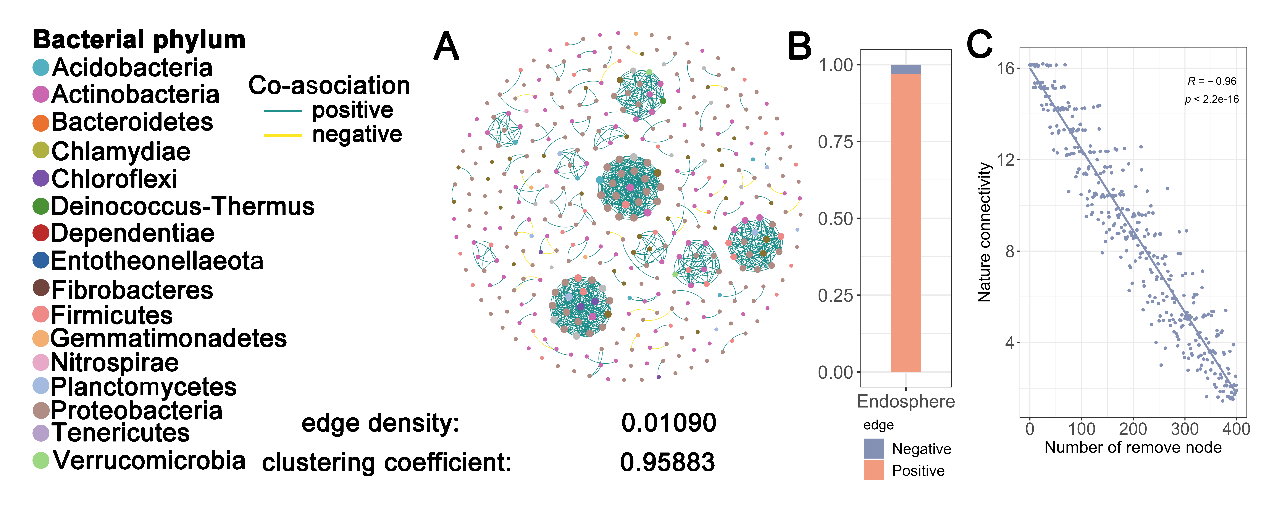


Figure S21 Complexity and stability of endosphere bacterial microbiota network under parasitism. A. Co-occurrence patterns of bacterial microbiota networks in the endosphere. B. The bar graph shows the proportion of positive and negative edges in endosphere microbiota network. C. The robustness of the microbiota networks was based on natural connectivity.
